# UPLC-MS/MS-Based Target Screening of 90 Phosphodiesterase Type 5 Inhibitors in 5 Dietary Supplements

**DOI:** 10.3390/molecules29153601

**Published:** 2024-07-30

**Authors:** Shaoming Jin, Yaonan Wang, Xiao Ning, Tongtong Liu, Ruiqiang Liang, Xinrong Pei, Jin Cao

**Affiliations:** 1 National Institute for Food and Drug Control, Beijing 100050, China; 2 School of Pharmaceutical Sciences, Capital Medical University, Beijing 100069, China

**Keywords:** target screening, UPLC-MS/MS, illegal addition, phosphodiesterase type 5 inhibitors

## Abstract

The aim of individuals consuming health supplements is to attain a robust state through nutritional regulation. However, some unscrupulous manufacturers, motivated by profit, fraudulently incorporate drugs or unauthorized components with therapeutic effects into the product for instant product performance enhancement. The long-term use of these products may inadvertently inflict harm on human health and fail to promote nutritive healthcare. The illegal inclusion of these substances is prevalent in kidney-tonifying and sexuality-enhancing products. Developing effective analytical methods to identify these products and screen for illegal added ingredients can effectively prevent such products from reaching and remaining on the market. A target screening method for the detection and quantification of 90 phosphodiesterase type 5 inhibitors (PDE-5is) in 5 kinds of health products was developed and validated. The type of dietary supplements varied from tablets, capsules, and protein powder to wine and beverages. Sample preparation was completed with a one-step liquid phase extraction. The screening process of 90 PDE-5is was done efficiently within 25 min by ultra-high performance liquid chromatography tandem mass spectrometry (UPLC-MS/MS) using the dynamic multiple reaction monitoring (dMRM) technique. The LODs of 90 PDE-5is were detected at levels ranging from 25 to 85 ng/g or ng/mL. This novel targeting methodology was effective and can be applied to routine market supervision. Among 286 batches of samples, 8 batches were found to be positive. Three kinds of PDE-5is were first detected in healthy products. The screening method demonstrated herein will be a promising and powerful tool for rapid screening of PDE-5is.

## 1. Introduction

The global trend toward dietary supplements to encourage healthier living has consistently intensified appreciably [1,2]. Considering the burgeoning demand for dietary supplements, potential risks also escalate concurrently. Throughout the previous decade, instances of suspected illegal adulterants of pharmaceuticals, unapproved drugs, and regulated substances have amplified markedly, posing a significant global challenge to the safety surveillance of dietary supplements [3]. Phosphodiesterase type 5 inhibitors (PDE-5is) can selectively inhibit the activity of the PDE-5 enzyme and increase the concentration of cyclic guanosine monophosphate (cGMP) in cells, resulting in smooth muscle relaxation [4]. At the earliest stage, it was used as a clinical drug for cardiovascular diseases [5]. Later, it was found that it has a therapeutic effect on patients with erectile dysfunction (ED), and nowadays, it has been used as the first-choice drug for the treatment of ED [6,7,8]. In pursuit of improving sexual ability, PDE-5is are often unethically added to various health products and dietary supplements. Most of these additions are unmarked and illegal, and taking these products without the guidance of a doctor is a great threat to health [9,10]. 

Several methods of PDE-5is detection or screening have been developed to effectively oversee and combat this illegal addition [11,12,13,14,15,16]. Most of these methods rely on liquid chromatography tandem mass spectrometry (LC-MS/MS) [17,18,19,20,21,22,23]. According to the differences in instruments, these methods are bifurcated into two main classifications: non-target screening methods based on high-resolution mass spectrometry and target screening methods based on tandem quadrupole mass spectrometry [24]. Non-target screening utilizes a database of precise reference material MS and MS/MS profiles acquired on high-resolution mass spectrometry [25]. The number of compounds screened hinges upon the contents of the database. Data processing consumes 70% of the whole screening work. Compound parameters, such as retention time and MS/MS spectrum are primary matching elements. Filtered parameters, such as the threshold of matching, are key factors affecting the screening results [26,27,28]. 

In contrast to the non-target methodology, the target screening approach is guided by optimizing acquisition parameters. The retention time and multiple reaction monitoring (MRM) transition channels of each compound have been fine-tuned to obtain the highest screening efficiency. The retention time and multiple reaction monitoring (MRM) transition channels for each compound have been meticulously curated to attain the utmost screening efficacy. The precise calibrators crafted by the reference materials are employed to construct the standard curve, facilitating not merely target screening but also precise quantification of the screened compounds [29]. Over half of the target screening endeavor entails sample preparation and data acquisition. The established screening methodology by reference material data allows for swift sample examination and compound quantification. False positive rates are significantly lower for target screening methods compared to non-target screening methods. For the scrutiny of health-threatening compounds, target screening methods with superior accuracy are typically employed. Lee et al. simultaneously identified 38 PDE-5is in illicit erectile dysfunction products [30]. Philippe et al. developed a prompt target assessment methodology for 71 active and 11 naturally occurring erectile dysfunction ingredients found in potentially tainted or counterfeit goods. Furthermore, they incorporated a high-resolution full scan procedure into this method, enabling subsequent identification via an untargeted approach to minimize the potential for false-positive outcomes [31]. With the notable advancement in chromatographic separation and mass spectrometry acquisition, over 150 compounds can be target screened in a single analysis [32]. Qie crafted a swift method for the simultaneous quantification of 160 drugs in urine or blood [33]. Employing the power of the scheduled MRM mode, Yin and colleagues presented an extensive multiresidue analytical methodology for 210 drugs in pork within a concise 20 min [34]. These case studies vividly underscore the broad application of the targeted screening strategy in food regulation.

To scrutinize all PDE-5is discovered, this research refined a target screening methodology to assess 90 of them (outlined in Table S1) across five food substrates or wholesome products, encompassing tablets, capsules, protein powder, healthful wine, and functional beverages. There was a significant incidence of PDE-5is incorporation into these wholesome products; this research approach streamlined existing laboratory methodologies and notably enhanced the food surveillance capabilities in the fight against illicit additions.

## 2. Results and Discussion

### 2.1. Extraction Solvent Optimization

Most PDE5-is were aromaticity compounds with weak polarity and limited solubility in pure water; organic solvents such as methanol, ethanol, acetone, and acetonitrile were often used for sample pre-treatment [35,36]. Within this study, methanol and acetonitrile were utilized for sample dilution or extraction; all PDE5-is were identifiable in these two solvents. However, in blank matrix-spiked samples, some compounds such as hydroxythiovardenafil exhibited splitting chromatographic peaks in acetonitrile, particularly evident in samples of wine and beverages; however, it was ameliorated when methanol was employed as solvent. The splitting chromatographic peak in acetonitrile is shown in the upper part of Figure S1, and the ameliorated peak in methanol is shown in the lower part of Figure S1.

### 2.2. LC–MS/MS Optimization

For liquid chromatography separation, methanol and 0.1% formic acid aqueous solution were selected as the mobile phase due to the enhanced solubility of PDE-5is in methanol. Initially, a 10% organic phase was maintained for 1 min to ensure robust retention of the sample on the chromatographic column; subsequently, the proportion of the organic phase was incrementally increased to facilitate sequential elution of the compound for analysis. The proportion of the organic phase was elevated to 100% until 19 min and sustained for 3 min to ensure complete elution of all substances in the sample without accumulation on the chromatographic column to prevent contamination. To optimally separate 90 PDE-5i, three distinct types of columns were employed. In comparison to HSS T3 (2.1 × 100 mm, 1.8 μm, Waters, Milford, MA, USA) and BEH C18 (2.1 × 100 mm, 1.7 μm, Waters, Milford, MA, USA), separation on Zorbax Eclipse Plus C18 (3.0 × 150 mm, 1.8 μm, Agilent, Santa Clara, CA, USA) was superior, as this column was the longest, exhibiting superior resolution for basic PDE-5is compounds. Figure S2 presents the outcomes of LC separation, employing three different chromatographic columns.

For MS detection, the unique standard solution (100 ng/mL) of each compound was administered into the mass spectrometer, and the fragmentation voltage was refined in full scan mode to facilitate the precursor ion to attain the utmost response. The polarities of the electrospray ionization (ESI) source were also optimized; all PDE-5is exhibited superior response in positive mode except depiperazinothio sildenafil, vardenafil dimer, and N-phenylpropylethyl tadalafil. These three PDE-5is manifested superior response in negative mode. Subsequently, the acquiring mode was shifted to the product ion mode to optimize the collision energy, and the two most abundant product ions were chosen for quantification and qualitative validation. Currently, PDE-5is primarily comprise two structural classifications, akin to the two popular sex-enhancing drugs presently available. The first comprises structural analogs of sildenafil, yielding fragment ions with a m/z of 311.1, 113.1, or 99.1; the second, structural prostaglandins of Tadalafil, predominantly produce fragment ions at a m/z of 135. The subtle differences in structure engender disparities in their collision energies, necessitating individual optimization.

**Figure 1:**
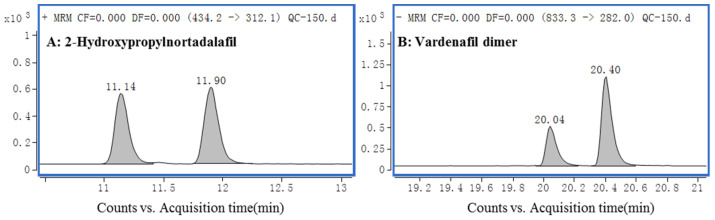
EICs of 2-hydroxypropylnortadalafil (**A**) and vardenafil dimer (**B**).

When all 180 transitions were configured in a single MRM method, the dwell time of each transition could only be allotted to 2 milliseconds when the cycle time was set to 400 milliseconds, which significantly impacted the sensitivity of the methodology. To mitigate this limitation, the dynamic multiple reaction monitoring (dMRM) modality was employed for data acquisition, wherein specific ion transitions were apprehended exclusively within a narrow retention time interval, thus potentially reducing the number of concurrent ion transitions and significantly augmenting sensitivity [37,38,39,40,41]. The extract ion chromatograms (EICs) of the transitions of all PDE-5is are shown in Figure S3. Among these 90 compounds, 2-hydroxypropylnortadalafil and the vardenafil dimer need special attention because they both have tautomerism; there were two peaks in the respective EICs, which are shown in Figure 1. The two peak areas need to be added together for quantification.

### 2.3. Selectivity, Linearity, Sensitivity, and Matrix Effects

The chromatograms of blank matrix and spiked matrix samples at a concentration of 100 ng/mL were juxtaposed to illustrate the selectivity of the devised methodology. The findings are illustrated in Figure S4. Peaks in the chromatograms of the blank matrix did not obstruct the analysis of PDE-5is in all five matrices, which validated the outstanding selectivity of the method.

The calibration curves of all 90 PDE-5is were in accordance with the quantitative analysis requirement, demonstrating excellent regression coefficient values exceeding 0.99. The matrix effects exhibited considerable fluctuations across the five diverse matrices. For tablets, healthful wine, and functional beverages, the matrix effects were within the range of 85–115%, indicating a negligible impact on PDE-5is quantification. For capsules and protein powder, the matrix effects were less than 80%, establishing matrix-matched calibration curves to mitigate the influence of the matrix. The regression coefficients of these two matrix-matched calibration curves were superior to 0.99, reflecting superb linearity. The method LODs and LOQs were evaluated utilizing the spiked-in sample in a blank matrix. The LOD and LOQ of each of the 90 PDE-5is are detailed in Table 1. The LODs of these 90 PDE-5is were less than 50 ng/g or 30 ng/mL. These thresholds were relatively low and suggest that the developed methodology is sufficiently sensitive to quantify the PDE-5is in various matrices.

### 2.4. Precision and Recoveries

The degrees of precision were computed through the ascertainment of mixed standard solutions spiked in 5 distinct blank matrices at concentrations of 15 ng/mL, 80 ng/mL, and 150 ng/mL. Six replicates of these solutions were quantified on a single day to assess intra-day precision, and the identical samples were assessed continually for three consecutive days to appraise the inter-day precision. All intra-day degrees of precision were below 10%, and all inter-day degrees of precision were below 15%. All recoveries of the solutions formulated at 3 concentrations in 5 matrices fell within the range of 80–120%. The mean precision and recoveries of the solutions in five matrices at divergent concentrations are illustrated in Table 2.

### 2.5. Method Application

The established method has played an important role in health product risk monitoring. From the perspective of product efficacy claims, samples can be segmented into five categories: boosting immunity, alleviating fatigue, reducing blood pressure, and amplifying sexual performance, and others. Within 286 cohorts of samples, PDE-5is were identified in 8 cohorts. Sildenafil was detected in 6 cohorts of tablet samples. It was a traditional illicit additive with a positive detection rate of 2.1%. In addition to 4 instances identified in samples that enhanced sexual efficacy, 2 events were identified in samples that mitigated fatigue. This suggests that illegal additions are becoming progressively more concealed, escalating the complexity of risk surveillance. 2-Hydroxypropyl nortadalafil was detected positively in one of the nutritionally beneficial wine samples. N-Ethyltadalafil was identified in one of the capsule samples. These two inhibitors have not been detected in our previous screening endeavors, signifying that the illegal incorporation of PDE-5is has become progressively covert. For all screening specimens, the overall positive detection rate was 2.8%; it is imperative to persistently expand the spectrum of detection compounds for food risk evaluation.

## 3. Materials and Methods

### 3.1. Reagents and Standards

The names, molecular formulas, and CAS numbers of the exceptional 90 PDE-5is standards are detailed in Table S1. Reference standards (purity ≥ 98%) for the 90 PDE-5is were obtained from Dr. Ehrenstorfer Laboratories (Augsburg, Germany). Formic acid (LC-MS grade) was purchased from Merck Co. (Darmstadt, Germany). Methanol (MeOH, LC-MS grade) was purchased from Honeywell Burdick & Jackson (Muskegon, MI, USA). Ultrapure water (18.2 MΩ) was obtained from a Milli-Q Advantage ultrapure water purification system. Healthy products such as vitality tablets, epimedium capsules, ginseng wine, protein power, and functional energy beverages were obtained from the National Institutes for Food and Drug Control (Beijing, China). 

### 3.2. Sample Preparation

Liquid substances, such as wine and beverages, were precisely determined after dilution to an exact volume. For solid substrates, the tablets were meticulously crushed into a uniform powder and blended thoroughly. The shells of all capsules were gently eliminated, and the powder within was delicately mixed. Typically, 1 g (±0.05 g) of solid samples (tablets, capsules, and powder) or 1 mL of liquid samples (wine and beverage) were precisely transferred into a 10 mL volumetric flask and dissolved in 7 mL methanol, followed by gentle sonication for 10 min. The volume of all samples was precisely fixed to 10 mL after the solid matter was completely dispersed or dissolved. Then, 5 mL methanol was transferred to a centrifuge tube and centrifuged at 14,000 rpm for 5 min sequentially; the supernatant was collected and filtered through a 0.22 μm filter (NormJect, Tuttlingen, Germany) prior to analysis. 

### 3.3. Preparation of Standard Solutions and Calibration Curve

Stock dilutions of the individual standards were meticulously prepared in methanol (ranging from 300 to 1000 μg/mL) and preserved at −80 °C. The working standard combination solution of 90 PDE-5is was formulated monthly at a concentration of 3 μg/mL in methanol and carefully stored at −20 °C. Blank instances were diligently processed, as outlined in Section 3.2, to yield a blank matrix solution. Subsequently, the matrix-matched calibration solution with the highest concentration (200 ng/mL) was prepared by fortifying the working solution (3 μg/mL) into a blank matrix solution. Finally, the remaining five matrix-matched calibration solutions were prepared by progressive dilution with the highest matrix-matched calibration solution (200 ng/mL). Mixed standard solutions with concentrations of 5, 10, 20, 50, 100, and 200 ng/mL were employed for constructing matrix-matched calibration curves. All calibrators were meticulously prepared prior to use. External standard calibration was utilized for the quantitative analysis.

### 3.4. LC-MS/MS Conditions

Chromatographic separation was carried out on an Agilent 1290 ultrahigh-performance liquid chromatography setup, employing an Agilent Eclipse Plus C18 column (3.0 × 150 mm, 1.8 μm) with the managerial column oven temperature preserved at 35 °C. The separation conditions underwent optimization: the infusion rate was set to 400 μL/min, the injection volume was 5 μL, and deionized water with 0.1% formic acid and methanol were the mobile phases A and B. A gradient routine was employed for elution: 0–1 min, 10% B, 1–16 min, 10–65% B, 16–19 min, 65–100% B, 19–22 min, 100%B, 22–22.1 min, returned to 10% B, and re-equilibrated for 3 min.

MS analysis was executed on an Agilent 6460 triple quadrupole (QQQ) mass spectrometer furnished with an AJS electrospray ionization source (ESI). The fine-tuned ESI conditions were: drying gas (N2) temperature at 200 °C, drying gas flow at 14 L/min, sheath gas (N2) temperature at 250 °C, sheath gas flow at 11 L/min, capillary at 3000 V, and nebulizer gas at 40 psi. The acquisition was conducted in dynamic multi-reaction mode (dMRM) with a cycle duration of 500 ms. Two specific transitions for each PDE-5i were monitored over an automatic set delta retention time. The detailed parameters of the established methodology are illustrated in Table S2.

### 3.5. Method Validation

The optimized sample screening methodology was meticulously validated in terms of selectivity, linearity, quantification, precision, recovery, and sensitivity in various matrices [30,35,36]. Selectivity was evaluated by comparing the chromatograms of 80 ng/mL standard analyte-spiked solutions and blank matrix, confirming the full resolution of all MRM extracted ion chromatograms (EICs) devoid of matrix interference. The peak area was correlated with specific spiked analyte concentrations within a blank matrix to formulate calibration curves. Calibration linearity was assessed using the coefficient of determination (R^2^). Accuracy and precision were confirmed through the analysis of spiked replicates at 3 concentration levels (15 ng/mL, 80 ng/mL, and 150 ng/mL), serving as quality control (QC) samples. Precision was expressed as a relative standard deviation (RSD, %) of the measured concentrations in spiked replicates. The recoveries of analytes across diverse matrices were also calculated at identical concentrations. The method’s reliability was gauged by establishing the limits of detection (LODs) and quantification (LOQs) of the spiked samples. When the signal-to-noise ratio (S/N) exceeding 3 for an individual target analyte in sequential dilutions was classified as LOD, it reached 10 signified LOQ. Matrix interference was assessed by comparing the measured peak area of analytes from QC samples to the predicted value using standards prepared in the solvent.

## 4. Conclusions

A targeted detection methodology for the concurrent quantification of 90 PDE-5is in five types of wholesome foods was efficiently designed and validated. The sample extraction procedures were straightforward and dependable, eliminating the necessity for a complementary clean-up protocol. The detection and quantitative evaluation of the 90 PDE-5is could be accomplished utilizing UHPLC-MS/MS, with a gradient chromatographic runtime of merely 25 min, executed in dMRM mode. This methodology provides an adept approach to target detection of PDE-5is in wholesome food with superior selectivity, precision, and sensitivity. Further expansion of this methodology could be achieved by augmenting the newly identified PDE-5i to enhance the capability of illicit addition detection.

## Figures and Tables

**Table 1:** The LODs and LOQs of 90 PDE-5is obtained in five different matrices.

Compound	Tablet /(ng/g)		Capsule /(ng/g)		Protein Powder /(ng/g)		Healthful Wine /(ng/mL)		Functional Beverage/(ng/mL)	
	LOD	LOQ	LOD	LOQ	LOD	LOQ	LOD	LOQ	LOD	LOQ
Sildenafil	47	162	75	198	79	246	52	155	35	101
Tadalafil	57	173	73	211	71	219	36	127	35	115
Imidazosagatriazinone	61	171	72	210	81	236	51	144	39	113
Gendenafil	56	174	73	234	79	254	47	147	27	102
Acetil acid	58	171	71	216	75	222	42	108	35	101
Xanthoanthrafil	45	147	67	237	75	205	38	123	25	95
Aminotadalafil	45	147	79	214	67	213	46	151	36	125
Chloropretadalafil	47	151	82	233	85	226	52	140	53	138
Piperiacetildenafil	52	162	66	205	76	240	40	125	44	114
Noracetildenafil	50	157	76	250	71	195	33	96	45	126
Carbodenafil	60	165	67	242	75	244	50	154	32	98
Pseudovardenafil	57	185	75	210	82	255	45	139	39	115
Norneosildenafil	51	145	80	237	79	252	42	119	35	115
N-Desethylvardenafil	55	173	80	238	71	213	38	117	45	143
N-Desmethylsildenafil	48	145	85	234	81	229	36	109	50	141
Acetildenafil	54	157	81	235	74	206	50	141	42	120
Hydroxyacetildenafil	63	179	76	205	80	231	44	134	36	107
Avanafil	47	146	72	209	69	221	54	144	33	98
Aildenafil	55	150	82	197	68	195	45	105	45	131
Homosildenafil	61	171	73	199	74	217	29	105	43	130
Vardenafil	59	179	79	238	72	220	48	141	38	123
Thiosildenafil	61	183	80	195	83	237	45	150	35	103
Thiohomosildenafil	56	171	77	235	75	205	37	119	36	112
Hydroxyvardenafil	50	145	80	212	79	242	52	137	42	124
Hydroxyhomosildenafil	60	175	82	201	84	246	41	130	44	125
Udenafil	65	181	72	222	69	220	35	125	41	126
Hydroxythiohomosildenafil	59	184	81	212	70	206	31	95	47	140
Norneovardenafil	50	150	65	195	77	238	38	105	51	153
Nitrodenafil	62	168	73	215	73	215	39	126	54	149
Nortadalafil	50	161	75	223	83	240	42	116	40	133
Chlorodenafil	60	171	83	241	79	234	45	129	52	153
Hydroxychlorodenafil	58	174	83	207	65	203	37	129	39	123
N-Butyltadalafil	64	182	70	245	72	225	47	147	35	126
Desmethylcarbodenafil	53	164	75	198	81	249	35	120	50	146
Descarbonsildenafil	47	154	69	205	78	233	53	154	38	124
Dimethylacetildenafil	62	175	73	232	73	195	29	104	35	120
Dithio-desmethylcarbodenafil	54	161	84	242	75	231	44	115	26	103
Oxohongdenafil	48	153	67	245	66	222	41	111	48	146
N-Octylnortadalafil	58	178	72	231	66	206	53	149	35	130
Dioxohongdenafil	55	176	84	204	81	251	28	105	45	117
Hydroxythiovardenafil	64	185	65	211	75	253	47	153	38	118
Cyclopentynafil	65	183	74	225	77	249	44	122	45	124
Propoxyphenyl thiohydroxyhomosildenafil	61	176	83	207	75	205	36	122	53	154
Benzylsildenafil	47	160	73	221	68	205	55	137	54	144
Cinnamyldenafil	61	170	72	210	75	211	45	138	35	105
Lodenafil carbonate	63	176	78	214	65	218	41	122	28	104
Propoxyphenylsildenafil	48	144	79	236	74	208	52	146	37	115
Depiperazinothiosildenafil	47	139	68	239	68	199	31	99	36	119
Acetaminotadalafil	46	136	76	197	69	223	36	115	46	140
2-Hydroxypropylnortadalafil	49	152	85	229	83	247	34	105	37	125
Acetylvardenafil	52	164	70	201	81	242	43	132	52	146
Propoxyphenyl hydroxyhomosildenafil	62	185	77	217	72	200	50	139	52	140
Propoxyphenyl thioaildenafil	58	176	69	248	83	251	51	145	45	125
Yohimbine	51	141	82	213	72	205	53	150	41	107
Dapoxetine	55	185	83	214	78	240	46	148	35	118
N-Desethylacetildenafil	45	159	80	212	71	213	41	128	39	116
Desmethylthiosildenafil	51	141	78	212	83	238	28	97	33	101
N-Boc-N-desethyl acetildenafil	61	167	71	201	66	217	38	122	35	114
N-Ethyltadalafil	46	155	82	215	72	216	41	115	39	125
O-Desethylsildenafil	60	173	79	212	72	222	28	105	41	117
Pyrazole N-desmethylsildenafil	49	153	75	252	76	246	34	100	29	101
Isobutylsildenafil	55	153	75	239	76	253	41	117	35	122
Sildenafil dimer impurity	61	183	76	230	81	245	30	103	54	146
Vardenafil oxopiperazine	45	151	70	204	65	209	43	115	38	115
Sildenafil N-oxide	59	183	78	208	78	228	53	135	37	128
Vardenafil N-oxide	50	139	77	195	77	245	33	99	42	124
2-Hydroxyethylnortadalafil	48	164	72	198	75	209	43	131	42	113
Vardenafil acetyl analogue	46	152	67	211	71	196	35	118	52	143
Vardenafil dimer	58	175	72	231	74	198	38	130	51	151
Mirodenafil	51	145	68	196	76	238	26	100	25	105
Mutaprodenafil	54	145	68	220	77	245	36	131	54	152
Thioquinapiperfil	63	174	80	214	78	241	45	145	31	100
Aminosildenafil	56	183	81	201	77	237	53	153	37	115
Desethylcarbodenafil	63	166	72	209	69	207	42	107	51	154
Didescarbonsildenafil	53	164	85	196	80	250	47	139	41	133
N-Phenylpropenyltadalafil	51	143	72	234	84	251	46	135	52	153
N-Desethyl-N-methylvardenafil	62	175	76	200	77	240	38	116	53	139
Thioaildenafil	63	172	74	209	71	207	46	148	30	99
Dichlorodenafil	48	158	84	230	65	224	36	107	25	95
Piperazonifil	64	168	83	253	83	236	35	108	44	105
Propoxyphenyl thiosildenafil	59	173	77	223	75	243	38	125	31	105
Propoxyphenyl thiohomosildenafil	46	146	83	195	72	225	37	126	45	109
Dithiodesethyl carbodenafil	47	149	76	220	74	196	43	129	49	145
Hydroxythioacetildenafil	65	185	69	215	85	239	46	145	36	111
Tadalafil dichloro impurity	51	154	79	234	79	227	35	112	37	107
Sildenafil impurity 12	52	156	85	231	80	236	36	127	35	115
Demethylpiperaziny sildenafil sulfonic acid	56	184	68	221	76	243	41	106	42	114
Propoxyphenyl aildenafil	47	141	75	233	78	226	55	146	38	106
Sildenafil impurity 14	53	135	82	253	69	202	38	133	37	121
Propoxyphenylisobutyl aildenafil	56	183	72	225	82	235	40	122	52	147

**Table 2:** The average recoveries of 90 PDE-5is obtained in five matrices (tablets, capsules, protein powder, healthful wine, and functional beverages) at 15 ng/mL, 80 ng/mL, and 150 ng/mL. The average inter-day precision was obtained using six replicates of the same solution as the recoveries mentioned above. The inter-day precision was obtained by continuous determination for three days of the same solution as the intra-day precision mentioned above.

Compound	Tablet/%			Capsule/%			Protein Powder/%			Healthful Wine/%			Functional Beverage/%		
	Average Recovery	Average Intra-Day Precision	Average Inter-Day Precision	Average Recovery	Average Intra-Day Precision	Average Inter-Day Precision	Average Recovery	Average Intra-Day Precision	Average Inter-Day Precision	Average Recovery	Average Intra-Day Precision	Average Inter-Day Precision	Average Recovery	Average Intra-Day Precision	Average Inter-Day Precision
Sildenafil	98.78	9.64	10.33	90.99	7.51	4.12	84.69	9.99	3.33	85.00	9.81	10.04	93.56	8.42	13.66
Tadalafil	85.17	5.42	11.68	95.33	6.91	9.19	86.45	6.38	8.52	107.05	7.68	9.78	94.13	4.65	5.18
Imidazosagatriazinone	96.40	2.48	4.14	96.20	8.40	11.53	89.94	8.52	9.22	100.74	6.46	12.44	91.11	3.24	13.02
Gendenafil	84.19	6.89	10.79	84.10	7.42	9.97	90.17	5.18	11.05	85.14	3.63	8.35	97.95	8.48	10.56
Acetil acid	86.97	9.35	3.17	84.07	8.10	4.96	87.87	6.25	6.15	102.58	8.52	11.09	97.16	5.91	6.87
Xanthoanthrafil	86.69	6.81	8.37	86.53	6.61	5.83	87.11	4.30	2.49	91.43	3.18	10.54	99.84	10.81	5.52
Aminotadalafil	90.06	7.01	8.64	93.21	4.20	2.33	81.32	5.15	1.72	103.70	2.98	6.36	92.80	9.94	10.50
Chloropretadalafil	96.57	10.50	11.56	91.66	2.40	7.67	91.88	7.18	7.52	81.12	8.83	9.32	91.18	8.70	5.79
Piperiacetildenafil	97.53	7.01	4.78	84.68	9.15	5.34	85.63	3.35	7.42	85.62	9.71	8.91	95.27	3.43	9.63
Noracetildenafil	97.00	9.05	3.91	82.91	7.49	1.02	91.66	4.53	2.66	104.24	6.50	6.92	92.49	9.06	12.67
Carbodenafil	94.67	6.16	5.82	84.01	8.33	12.08	91.77	4.50	4.05	101.60	4.63	6.32	92.25	3.99	11.13
Pseudovardenafil	98.90	6.63	6.57	80.02	6.93	5.83	84.21	9.10	3.53	98.73	3.17	10.10	91.05	8.43	11.20
Norneosildenafil	95.60	7.84	3.12	84.02	8.66	6.00	86.44	9.28	9.17	101.63	10.72	12.90	94.66	6.74	10.19
N-Desethylvardenafil	101.80	2.42	7.97	82.11	7.61	5.65	88.42	9.29	2.81	96.96	5.12	5.42	95.29	4.82	7.18
N-Desmethylsildenafil	99.98	9.86	7.83	94.37	5.35	9.80	85.31	2.63	7.05	86.36	9.76	6.64	96.94	3.83	10.39
Acetildenafil	102.85	8.65	9.37	93.10	9.38	9.99	87.58	10.87	6.38	83.18	4.04	7.96	93.52	5.62	11.59
Hydroxyacetildenafil	89.55	9.48	9.17	86.14	2.35	4.79	85.57	4.55	11.82	101.88	7.58	7.69	102.83	6.85	5.75
Avanafil	96.93	5.75	11.40	82.27	9.78	5.04	86.89	8.14	6.17	110.99	6.70	10.16	92.67	4.10	8.21
Aildenafil	100.11	3.86	3.69	92.31	6.69	5.23	85.97	4.88	11.88	103.62	7.08	8.40	95.53	3.81	11.04
Homosildenafil	88.95	8.00	2.37	80.89	5.51	10.40	81.12	7.39	7.79	82.45	9.09	13.10	91.54	10.84	13.71
Vardenafil	97.48	9.79	1.03	96.38	4.05	9.06	90.28	10.29	2.40	81.81	3.59	7.48	98.52	4.36	12.73
Thiosildenafil	99.34	10.68	11.87	84.71	8.53	5.24	82.04	2.32	10.94	84.58	5.08	6.70	91.96	7.38	10.63
Thiohomosildenafil	91.28	6.29	6.41	89.29	9.37	3.67	87.76	3.24	7.93	98.28	7.31	7.32	96.67	5.74	11.33
Hydroxyvardenafil	98.94	3.19	7.70	80.40	6.24	5.03	89.15	6.21	4.91	84.78	3.40	8.55	97.52	9.83	9.58
Hydroxyhomosildenafil	85.07	2.04	6.27	95.43	7.34	4.93	87.50	8.25	11.75	89.32	5.99	9.57	95.33	3.72	5.79
Udenafil	102.39	6.34	3.88	94.36	7.61	3.01	89.95	4.58	8.24	100.37	8.99	9.14	103.10	3.22	8.33
Hydroxythiohomosildenafil	104.84	10.47	1.67	87.76	7.71	5.75	87.12	10.22	5.71	111.52	7.83	12.01	100.81	6.64	9.74
Norneovardenafil	100.86	7.39	9.21	83.74	6.41	9.95	82.17	5.97	10.72	104.30	9.34	9.23	104.65	6.74	6.80
Nitrodenafil	80.70	7.06	3.11	82.86	7.58	3.24	83.02	7.91	10.69	85.49	6.17	5.06	99.17	6.11	9.44
Nortadalafil	99.35	4.59	10.54	87.60	6.39	12.13	81.85	6.59	7.09	84.86	5.61	7.32	101.35	5.93	12.63
Chlorodenafil	97.79	2.67	7.79	85.89	8.13	3.79	86.07	6.54	5.60	109.57	8.42	13.88	100.99	7.54	11.78
Hydroxychlorodenafil	87.41	3.87	5.02	82.12	2.36	6.10	91.05	3.33	2.90	90.36	4.98	9.97	104.75	3.52	9.02
N-Butyltadalafil	94.32	9.84	11.78	91.10	2.51	6.98	90.65	8.44	7.46	87.09	3.61	14.12	98.90	8.01	8.57
Desmethylcarbodenafil	86.97	9.52	7.36	86.39	7.26	7.06	89.82	6.61	2.93	93.17	5.80	12.32	99.43	3.94	7.51
Descarbonsildenafil	89.39	2.62	7.97	89.06	8.59	1.17	82.47	10.48	5.55	88.76	5.48	5.10	106.53	4.96	9.14
Dimethylacetildenafil	104.06	4.01	12.38	86.55	8.10	4.34	90.96	4.95	3.54	100.19	4.76	13.35	103.16	8.47	9.03
Dithio-desmethylcarbodenafil	97.97	5.24	9.29	81.68	6.34	8.25	86.80	3.78	8.34	84.31	3.91	11.67	100.93	6.40	11.36
Oxohongdenafil	96.19	5.13	3.74	83.73	7.07	8.89	92.42	9.25	4.52	80.36	3.88	9.59	101.95	7.86	6.26
N-Octylnortadalafil	100.63	8.45	6.67	95.72	4.28	4.03	83.01	8.20	8.32	99.04	7.42	8.56	98.03	2.28	8.33
Dioxohongdenafil	95.99	8.20	8.32	94.20	6.52	5.67	87.31	5.02	5.57	80.51	8.07	8.27	97.18	4.93	7.27
Hydroxythiovardenafil	105.12	8.59	2.96	85.34	8.27	3.28	83.69	5.10	5.87	107.10	9.40	11.81	100.68	9.31	9.33
Cyclopentynafil	89.98	7.77	11.69	91.35	8.26	9.87	90.24	5.97	6.95	102.71	8.89	12.44	99.07	3.83	14.17
Propoxyphenyl thiohydroxyhomosildenafil	96.08	6.65	12.60	95.53	5.31	6.86	86.28	5.21	10.89	96.16	3.57	13.10	106.46	8.42	8.98
Benzylsildenafil	101.29	7.35	2.40	82.77	2.34	7.96	90.30	7.46	2.93	94.17	9.28	7.22	100.30	5.72	13.26
Cinnamyldenafil	101.94	1.30	2.92	82.41	5.22	9.30	86.77	8.95	10.34	110.82	4.41	6.59	108.06	10.55	13.55
Lodenafil carbonate	100.28	1.41	9.38	81.20	6.32	10.98	89.55	10.38	7.85	84.09	3.75	12.78	101.63	4.18	7.11
Propoxyphenylsildenafil	97.57	3.20	6.49	85.20	5.27	11.10	85.09	6.40	7.39	105.78	4.70	7.04	102.19	7.97	8.43
Depiperazinothiosildenafil	87.90	7.91	9.29	81.04	9.21	4.85	88.15	6.63	3.36	100.47	7.91	14.12	99.67	8.34	13.16
Acetaminotadalafil	85.68	2.70	3.36	82.82	3.23	10.76	93.00	7.03	3.04	87.72	4.39	5.46	99.46	8.32	13.76
2-Hydroxypropylnortadalafil	104.07	0.67	3.82	86.83	1.50	5.32	81.86	4.39	2.69	102.50	6.18	9.64	102.94	7.26	8.71
Acetylvardenafil	96.46	3.51	6.15	85.10	6.98	5.99	84.33	9.20	2.58	82.55	3.03	9.23	101.08	5.89	9.89
Propoxyphenyl hydroxyhomosildenafil	95.63	5.47	2.95	89.97	5.39	10.68	81.41	6.16	8.81	89.53	4.15	10.10	104.90	3.70	8.03
Propoxyphenyl thioaildenafil	96.75	5.42	9.04	94.84	2.64	2.69	83.97	5.92	0.27	111.05	10.98	12.31	107.62	6.60	9.81
Yohimbine	95.43	4.25	9.01	85.40	1.41	9.58	84.73	8.20	3.47	81.94	4.51	9.71	87.40	7.89	6.36
Dapoxetine	81.95	3.65	6.83	95.08	8.16	11.45	86.48	9.49	6.58	96.19	5.24	10.83	106.54	7.36	12.59
N-Desethylacetildenafil	100.24	8.21	4.29	95.38	8.86	7.86	81.09	8.20	10.92	101.26	7.09	8.84	95.56	8.14	14.04
Desmethylthiosildenafil	103.95	2.09	6.60	81.94	5.12	9.18	85.50	8.61	1.74	92.61	5.55	7.96	108.09	9.46	13.57
N-Boc-N-desethyl acetildenafil	90.85	7.08	10.76	95.39	3.03	9.34	87.91	3.10	8.20	90.05	4.41	11.50	101.58	2.10	12.24
N-Ethyltadalafil	91.42	8.78	6.50	83.17	6.08	10.55	89.66	6.08	5.22	83.45	3.63	8.39	98.56	3.78	14.66
O-Desethylsildenafil	102.51	8.72	11.60	94.87	6.08	8.38	91.35	4.95	2.84	93.78	9.19	12.00	96.69	3.09	13.27
Pyrazole N-desmethylsildenafil	102.08	5.44	11.59	80.02	1.14	7.98	91.27	5.44	9.75	97.77	5.38	10.00	99.01	2.78	13.12
Isobutylsildenafil	97.50	8.29	8.68	81.99	3.88	5.66	90.05	4.27	7.31	82.91	9.40	5.91	102.93	9.33	6.74
Sildenafil dimer impurity	100.04	3.65	10.93	83.95	1.58	5.36	84.07	9.24	11.47	88.43	9.23	11.19	98.96	8.03	10.68
Vardenafil oxopiperazine	97.74	3.80	8.66	93.22	6.65	9.13	89.28	9.64	1.12	101.69	5.27	13.16	99.65	4.40	6.20
Sildenafil N-oxide	98.83	7.67	4.92	82.12	4.86	7.78	90.96	6.56	11.81	109.34	10.96	7.58	97.57	8.73	6.33
Vardenafil N-oxide	89.97	8.80	8.46	82.65	10.36	7.46	87.48	9.58	7.69	103.99	8.62	9.17	84.97	8.11	14.85
2-Hydroxyethylnortadalafil	87.11	2.00	8.57	93.59	3.42	9.55	87.00	5.34	11.41	85.91	4.64	6.43	99.14	3.78	7.74
Vardenafil acetyl analogue	96.47	5.42	4.15	92.59	6.84	10.40	88.88	3.31	3.22	82.88	3.27	11.77	102.88	7.30	13.97
Vardenafil dimer	93.80	2.10	7.55	80.15	5.20	5.42	84.29	6.79	8.91	82.69	10.84	8.85	100.76	9.25	9.18
Mirodenafil	100.79	8.42	3.38	89.37	5.65	6.60	86.07	3.92	6.64	81.09	3.32	9.68	105.41	3.68	13.96
Mutaprodenafil	93.65	6.05	7.09	90.88	7.45	6.67	89.37	10.04	3.68	105.43	2.83	11.13	107.85	3.96	12.50
Thioquinapiperfil	84.85	7.50	10.20	87.74	4.64	4.18	89.31	10.80	6.90	83.93	9.10	7.15	94.07	7.74	12.15
Aminosildenafil	93.25	2.27	10.32	87.22	9.88	5.27	85.81	4.94	6.83	98.06	3.59	5.51	95.00	7.56	12.01
Desethylcarbodenafil	83.86	10.47	7.45	87.14	6.87	4.14	89.72	7.98	7.56	87.47	5.99	8.11	100.23	4.99	13.78
Didescarbonsildenafil	92.11	0.11	5.75	82.77	8.20	8.46	89.87	7.85	5.85	88.70	9.99	12.15	101.94	8.86	9.45
N-Phenylpropenyltadalafil	87.50	10.34	10.34	86.05	9.30	6.72	82.26	5.03	8.40	96.80	3.51	5.43	96.58	9.04	7.97
N-Desethyl-N-methylvardenafil	87.21	6.73	4.12	81.82	5.39	7.13	82.49	9.99	3.09	89.76	10.97	11.08	98.92	4.58	5.93
Thioaildenafil	91.19	1.71	9.48	83.48	6.33	5.62	88.65	6.27	9.71	93.20	7.81	5.08	105.73	6.45	8.99
Dichlorodenafil	93.00	8.81	9.15	95.95	9.24	3.80	92.05	3.55	2.17	92.10	8.55	6.19	106.93	4.60	10.32
Piperazonifil	91.11	6.46	7.86	90.37	8.23	12.49	84.64	6.71	9.85	94.17	7.13	13.74	100.26	3.93	7.41
Propoxyphenyl thiosildenafil	85.71	4.29	7.71	86.25	5.40	10.25	91.40	9.91	3.68	88.44	6.82	10.68	83.40	2.13	10.85
Propoxyphenyl thiohomosildenafil	84.74	6.36	3.16	89.17	7.65	7.77	83.32	7.73	2.64	99.30	4.92	9.65	108.86	10.76	11.14
Dithiodesethyl carbodenafil	82.64	7.81	10.79	85.32	5.98	5.39	83.78	7.07	4.28	80.85	4.62	8.18	96.97	4.45	13.02
Hydroxythioacetildenafil	98.44	4.41	6.97	96.25	4.25	5.39	81.42	6.51	11.12	93.00	10.92	12.58	98.76	4.77	6.29
Tadalafil dichloro impurity	95.27	3.26	10.87	93.53	7.21	10.25	83.19	4.09	2.37	87.54	10.31	14.15	102.20	3.41	8.02
Sildenafil impurity 12	103.09	0.94	6.40	85.95	9.22	9.86	83.80	9.51	12.53	92.33	5.80	11.36	103.12	8.02	11.72
Demethylpiperaziny sildenafil sulfonic acid	101.10	1.94	5.46	94.76	5.00	4.44	85.44	5.11	1.37	99.90	4.97	13.48	90.00	8.02	11.20
Propoxyphenyl aildenafil	96.14	5.93	11.30	86.75	10.77	6.70	90.99	10.07	1.35	92.08	6.08	13.29	97.73	9.07	10.56
Sildenafil impurity 14	91.79	10.16	10.41	82.57	3.13	7.72	88.25	3.28	4.20	95.07	9.10	12.92	89.28	10.24	10.71
Propoxyphenylisobutyl aildenafil	95.72	8.18	5.65	92.06	4.97	9.05	90.02	2.25	7.73	91.62	10.73	7.99	101.90	6.80	6.89

## Data Availability

The original contributions presented in the study are included in the article (and Supplementary Materials), further inquiries can be directed to the corresponding authors.

## Supplementary Materials

The following supporting information can be downloaded at: https://www.mdpi.com/article/10.3390/molecules29153601/s1.
