# Supplementary material for: UPLC-MS/MS-Based Target Screening of 90 Phosphodiesterase Type 5 Inhibitors in 5 Dietary Supplements
Source: Molecules. 2024 Jul 30;29(15):3601. doi: 10.3390/molecules29153601 (PMC11313696; doi:10.3390/molecules29153601)
Supplement: Supplementary file 1 [file molecules-29-03601-s001.zip › Supplementary figures mentioned in the manuscript.pdf]

UPLC-MS/MS Based Target Screening of 90 Phosphodiesterase Type 5 Inhibitors in 5 Species Dietary Supplements

Shaoming Jin [1,†], Yaonan Wang [2,†], Xiao Ning [1], Tongtong Liu [1], Ruiqiang Liang [1] , Xinrong Pei [1,*] and Jin Cao [1,*]

[1] National Institute for Food and Drug Control, Beijing 100050, China; myjackyming@126.com (S.J.); 15905171365@163.com (T.L.); nx200730079@163.com (X.N.); liangruiqiang1990@126.com (R.L.)

[2] School of Pharmaceutical Sciences, Capital Medical University, Beijing 100069, China; wangyaonan@ccmu.edu.cn

* Correspondence: peixinrong@nifdc.org.cn (X.P.), caojin@nifdc.org.cn (J.C.); Tel.: +86-010-6709-5264 (X.P.), +86-010-6709-5070 (J.C.)

† These authors contributed equally to this work.

Figure S1. The chromatographic peaks of hydroxythiovardenafil in different sample dilution solvent. 1

Figure S2. The LC separation results of three different columns. 2

Figure S3. The extract ion chromatograms (EICs) of transitions of all the PDE-5is. 2

Figure S4. The total ion chromatograms (TIC) of blank pill matrix sample (upper) and spiked-in sample in pill matrix at concentration of 80ng/mL (lower). 11

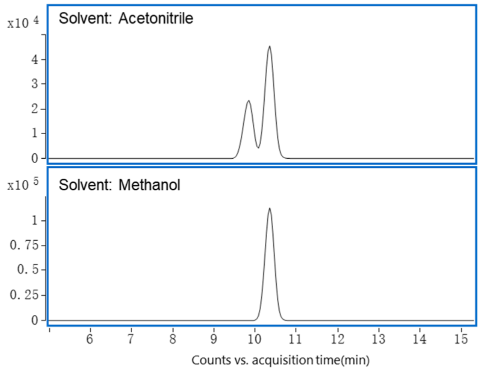

Figure S1. The chromatographic peaks of different sample dilution solvent.

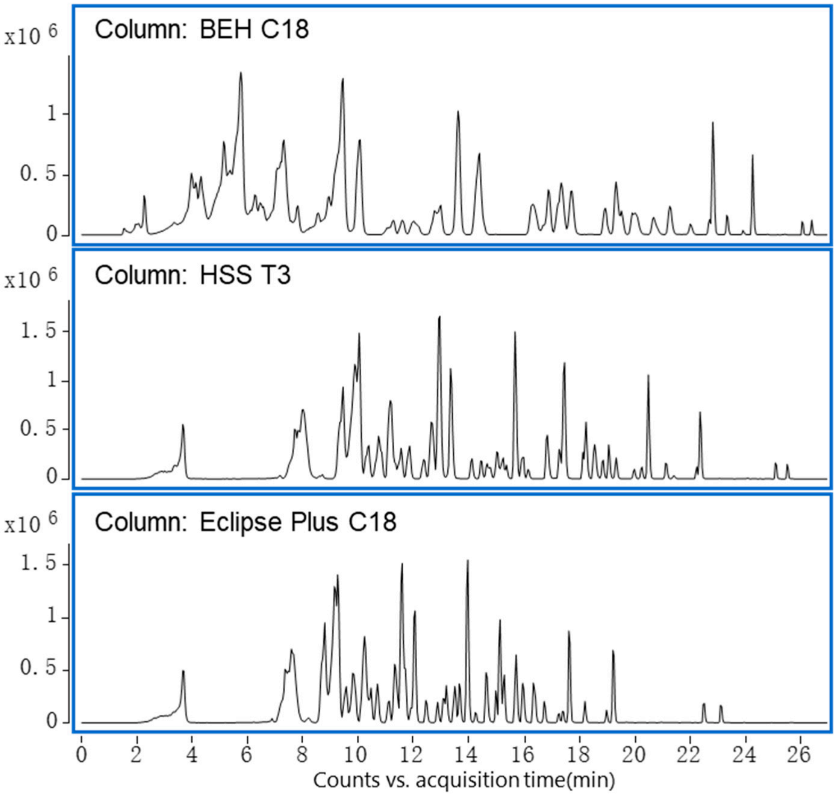

Figure S2. The LC separation results of three different columns.

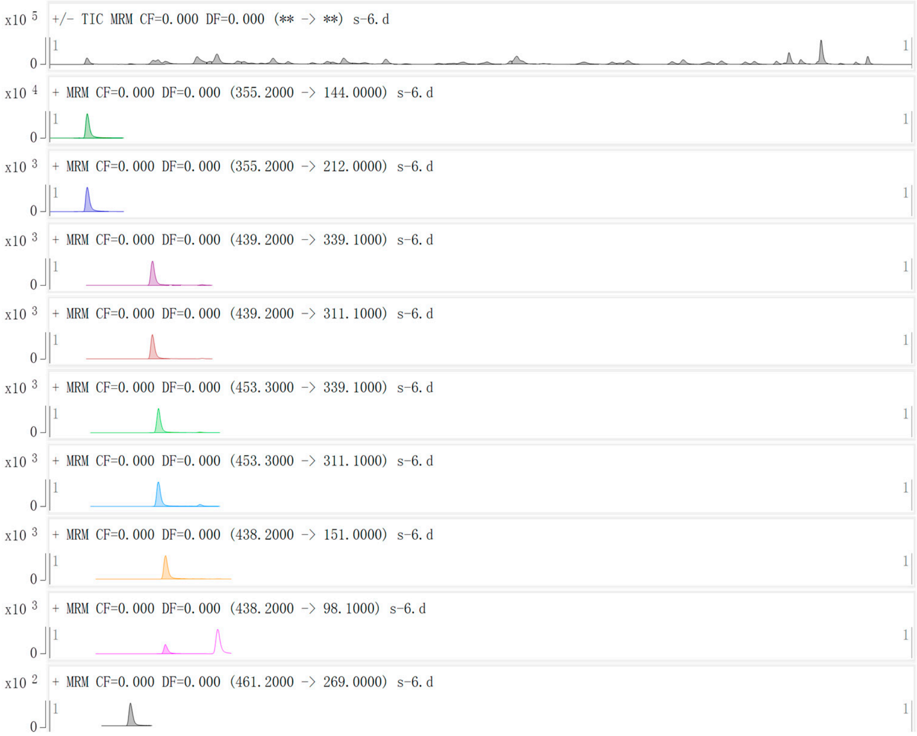

Figure S3. The extract ion chromatograms (EICs) of transitions of all the PDE-5is.

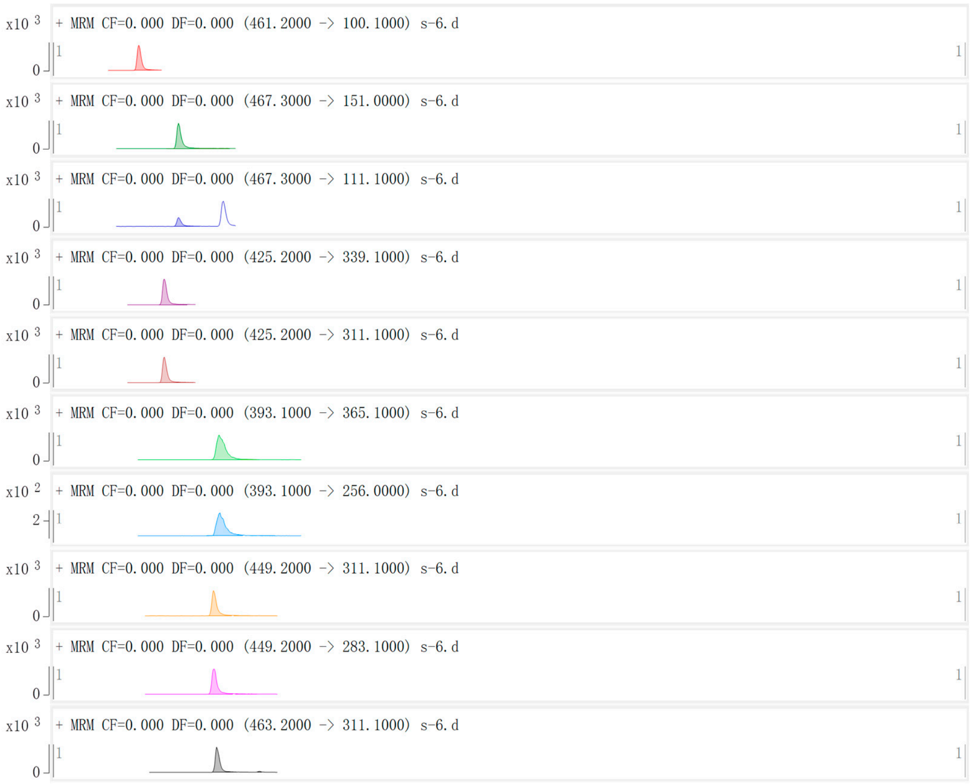

Figure S3-continued-1. The extract ion chromatograms (EICs) of transitions of all the PDE-5is.

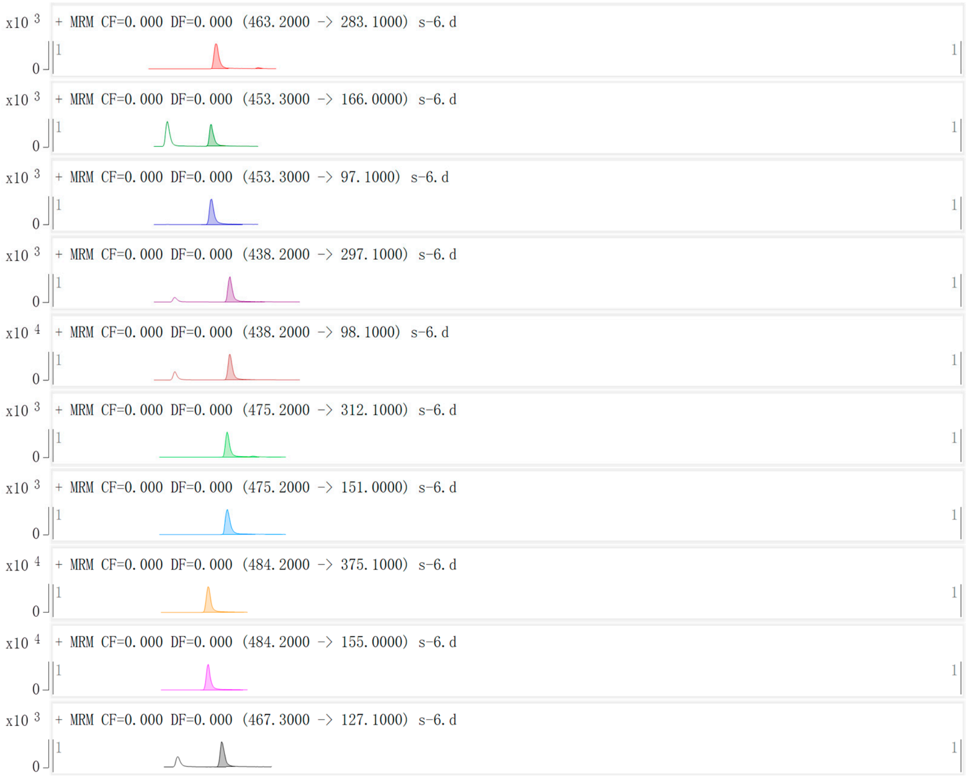

Figure S3-continued-2. The extract ion chromatograms (EICs) of transitions of all the PDE-5is.

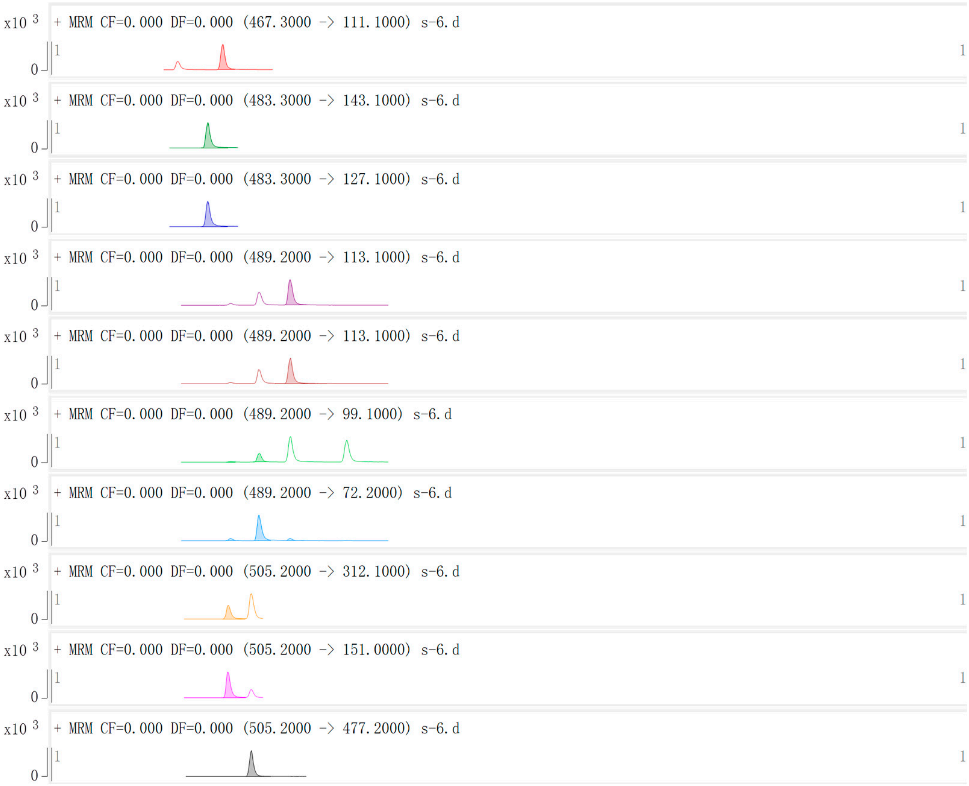

Figure S3-continued-3. The extract ion chromatograms (EICs) of transitions of all the PDE-5is.

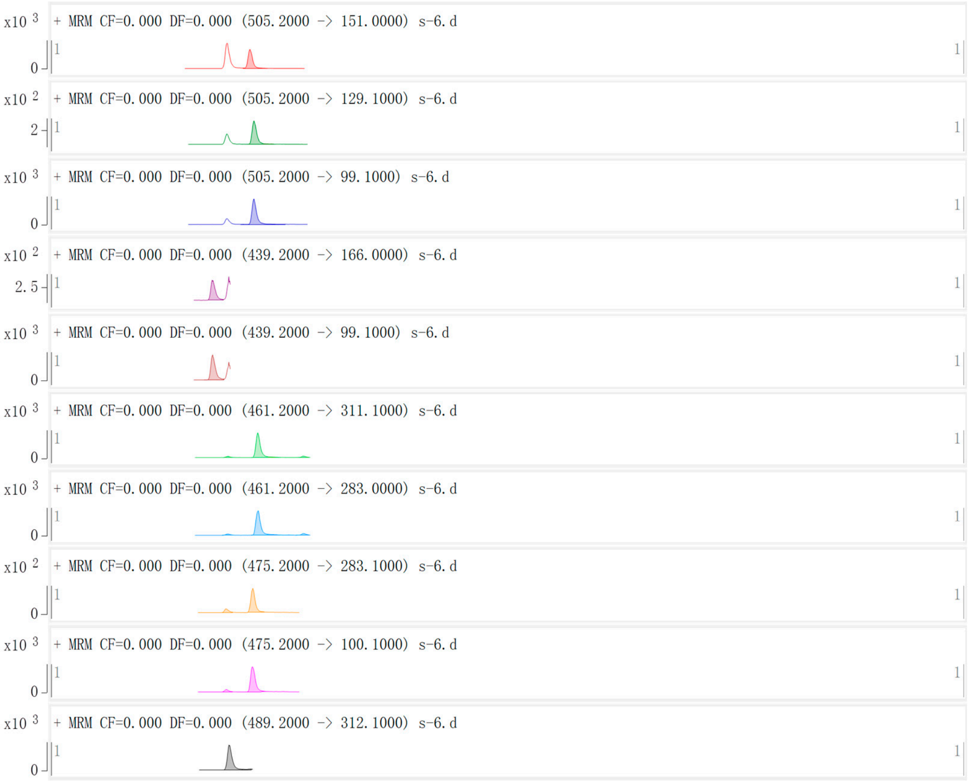

Figure S3-continued-4. The extract ion chromatograms (EICs) of transitions of all the PDE-5is.

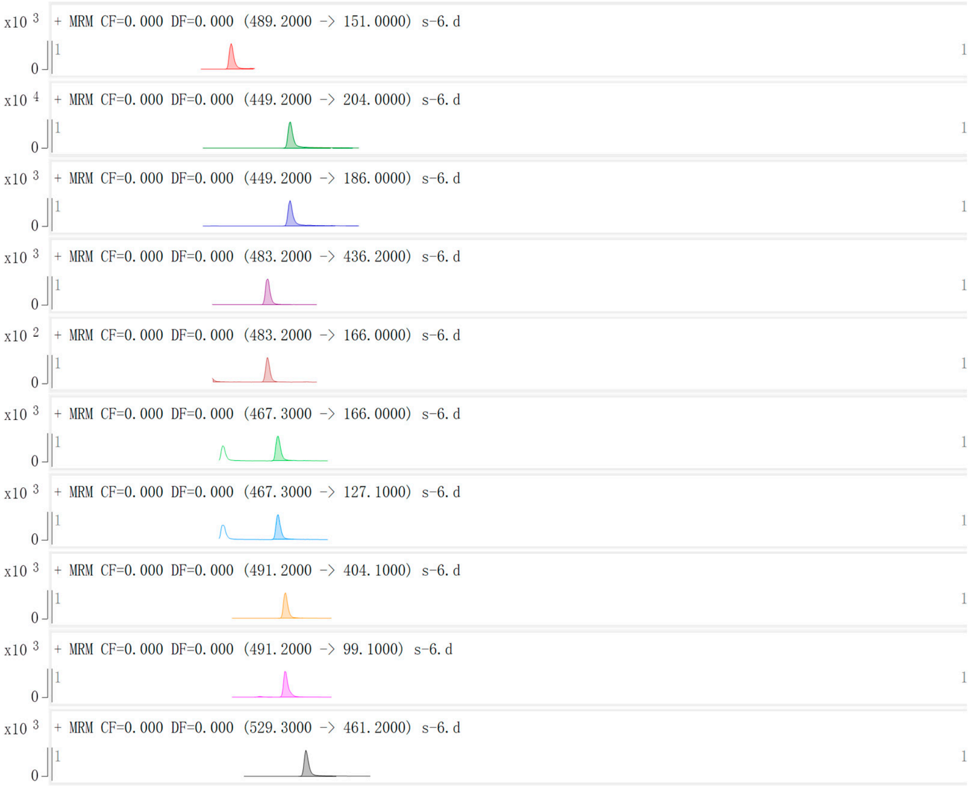

Figure S3-continued-5. The extract ion chromatograms (EICs) of transitions of all the PDE-5is.

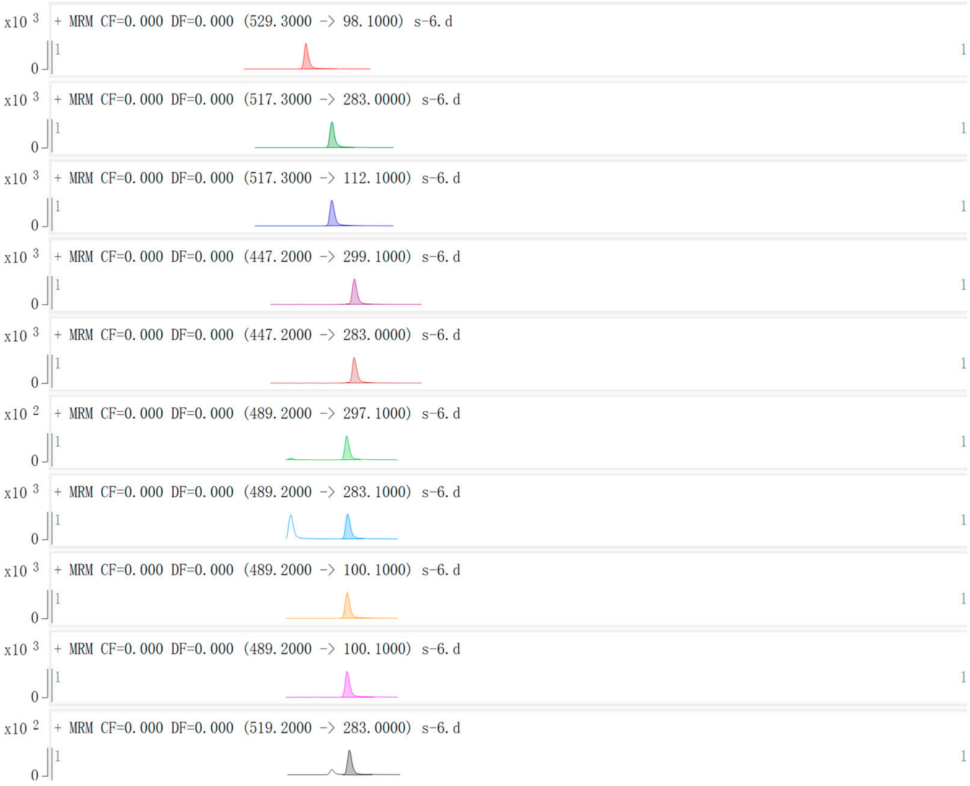

Figure S3-continued-6. The extract ion chromatograms (EICs) of transitions of all the PDE-5is.

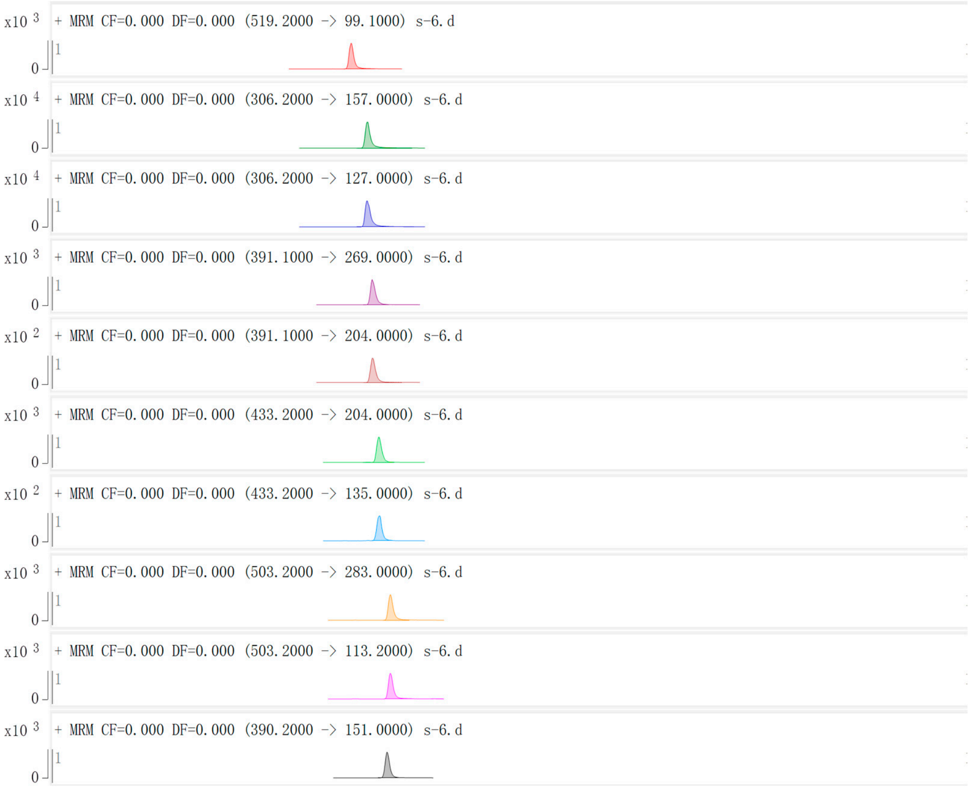

Figure S3-continued-7. The extract ion chromatograms (EICs) of transitions of all the PDE-5is.

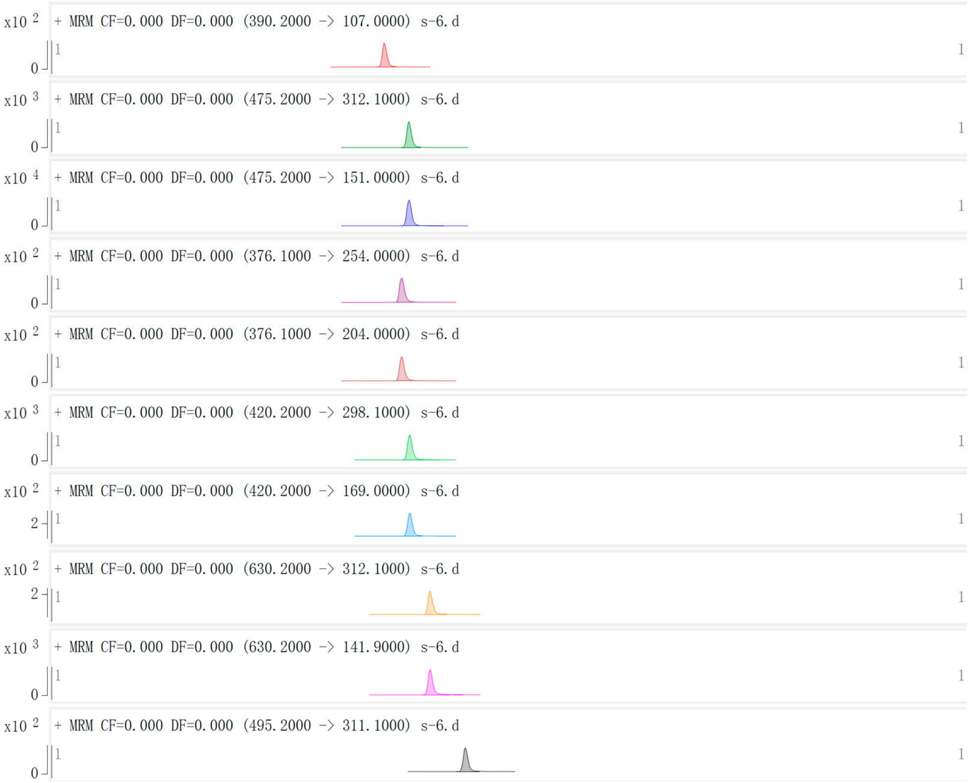

Figure S3-continued-8. The extract ion chromatograms (EICs) of transitions of all the PDE-5is.

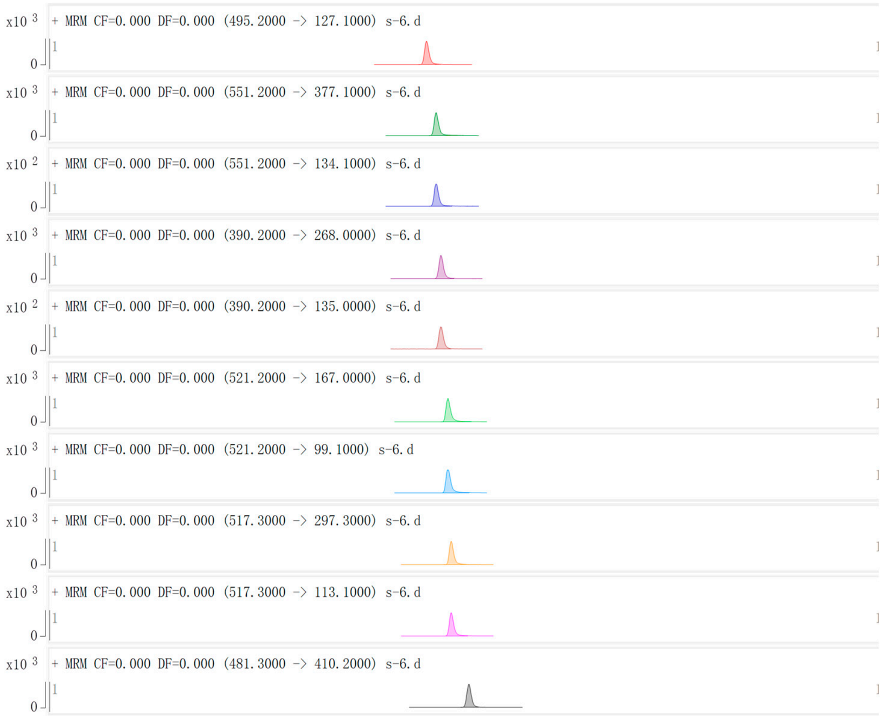

Figure S3-continued-9. The extract ion chromatograms (EICs) of transitions of all the PDE-5is.

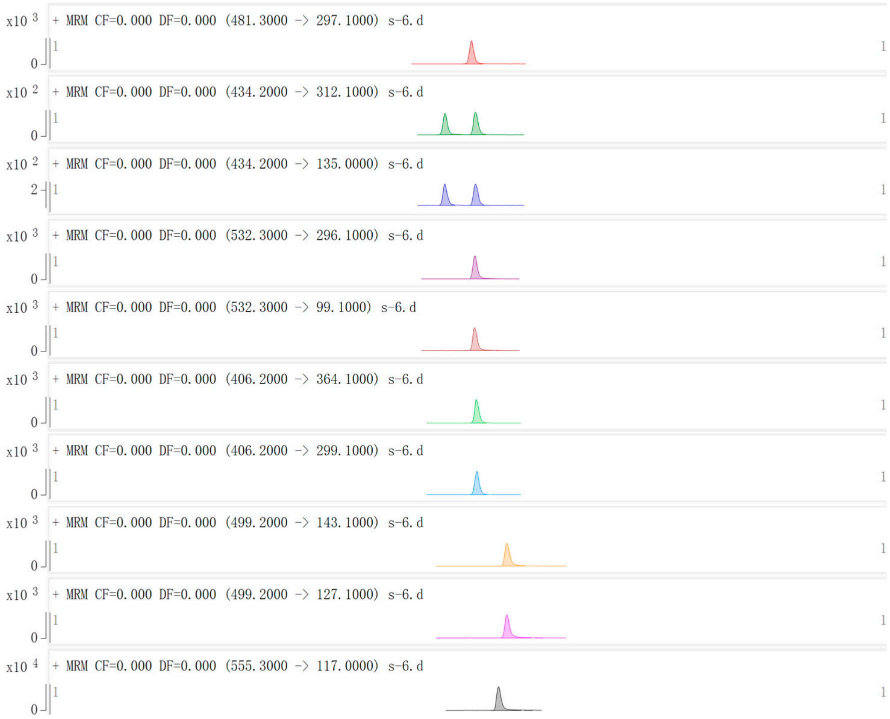

Figure S3-continued-10. The extract ion chromatograms (EICs) of transitions of all the PDE-5is.

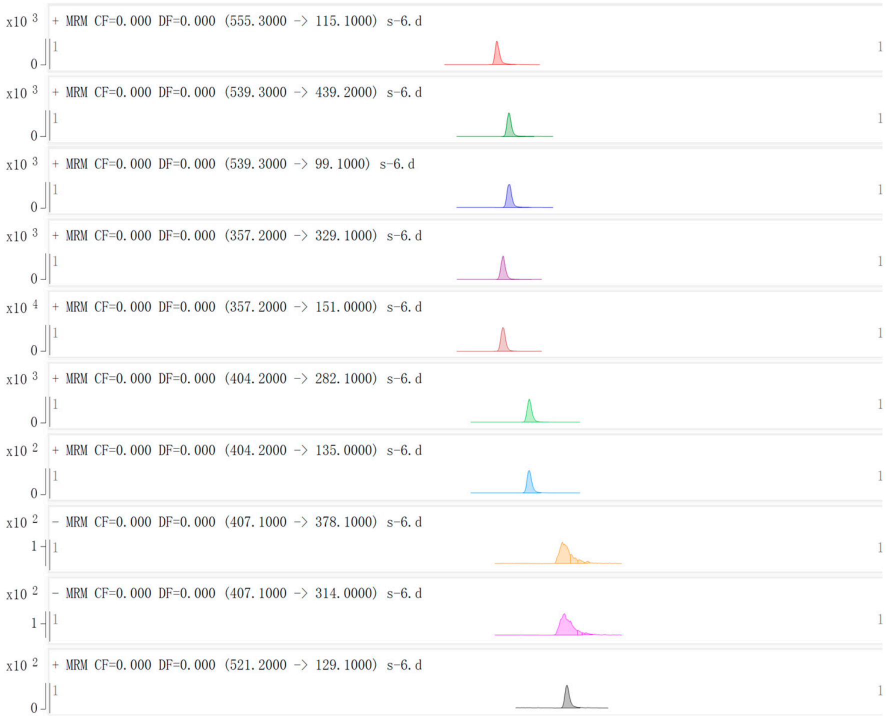

Figure S3-continued-11. The extract ion chromatograms (EICs) of transitions of all the PDE-5is.

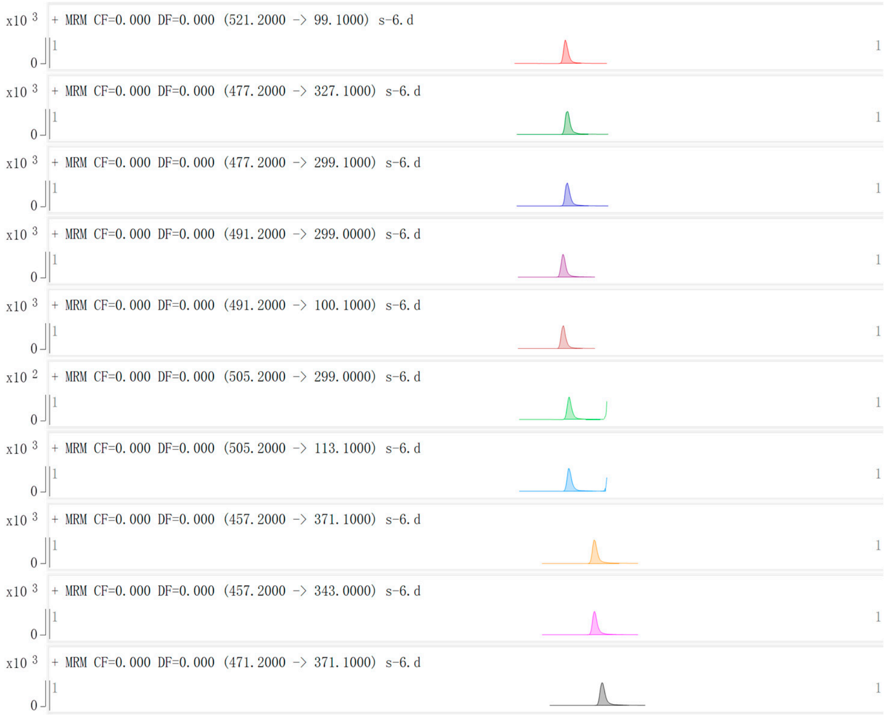

Figure S3-continued-12. The extract ion chromatograms (EICs) of transitions of all the PDE-5is.

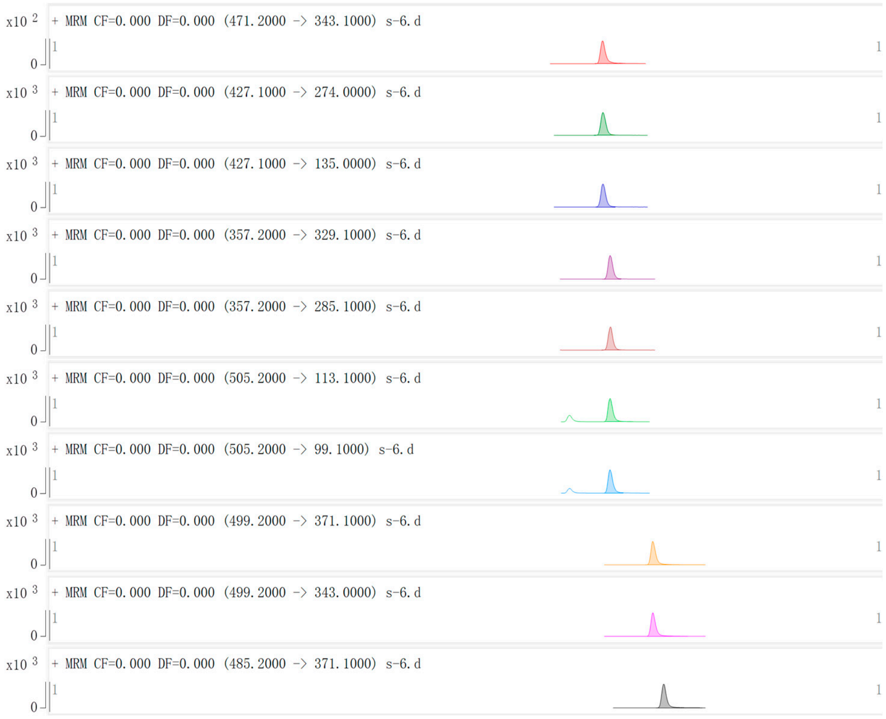

Figure S3-continued-13. The extract ion chromatograms (EICs) of transitions of all the PDE-5is.

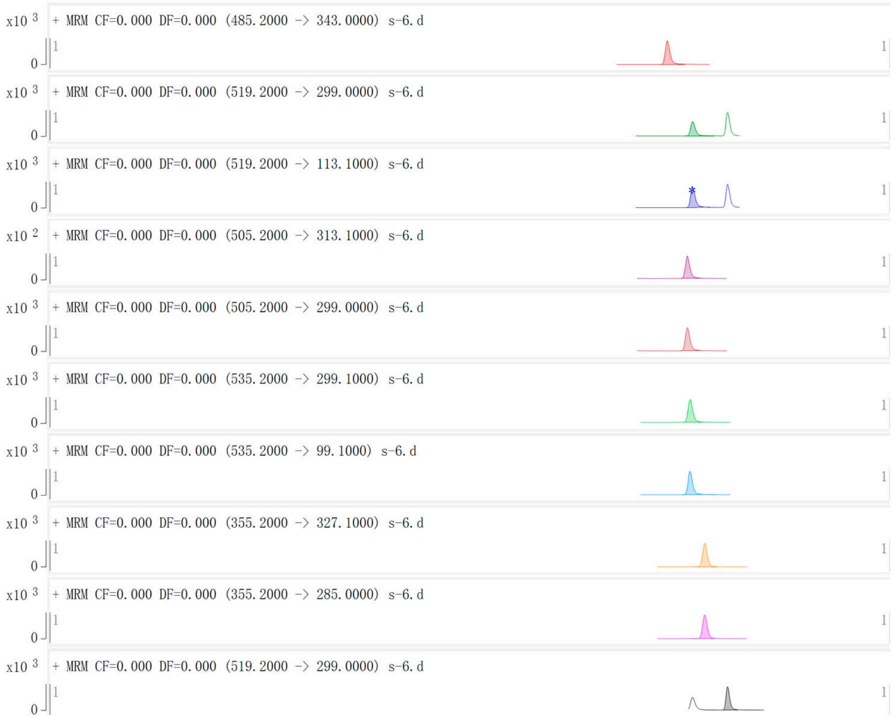

Figure S3-continued-14. The extract ion chromatograms (EICs) of transitions of all the PDE-5is.

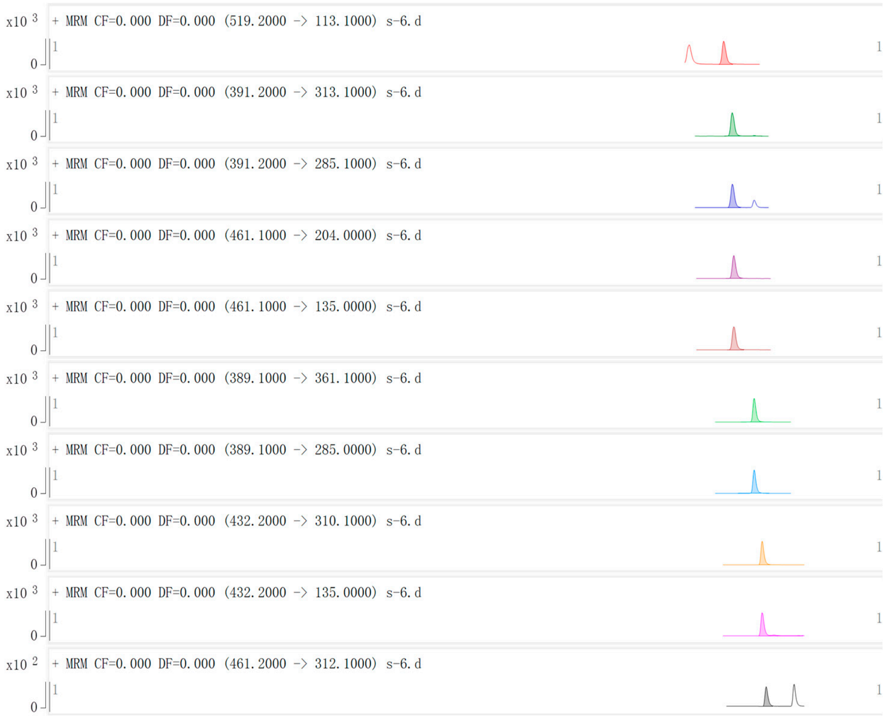

Figure S3-continued-15. The extract ion chromatograms (EICs) of transitions of all the PDE-5is.

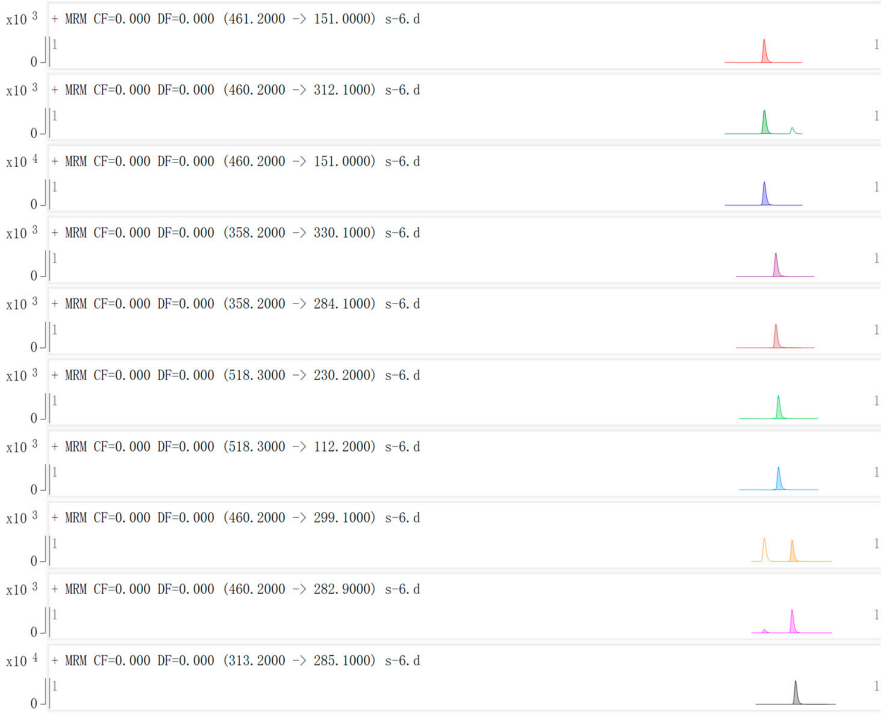

Figure S3-continued-16. The extract ion chromatograms (EICs) of transitions of all the PDE-5is.

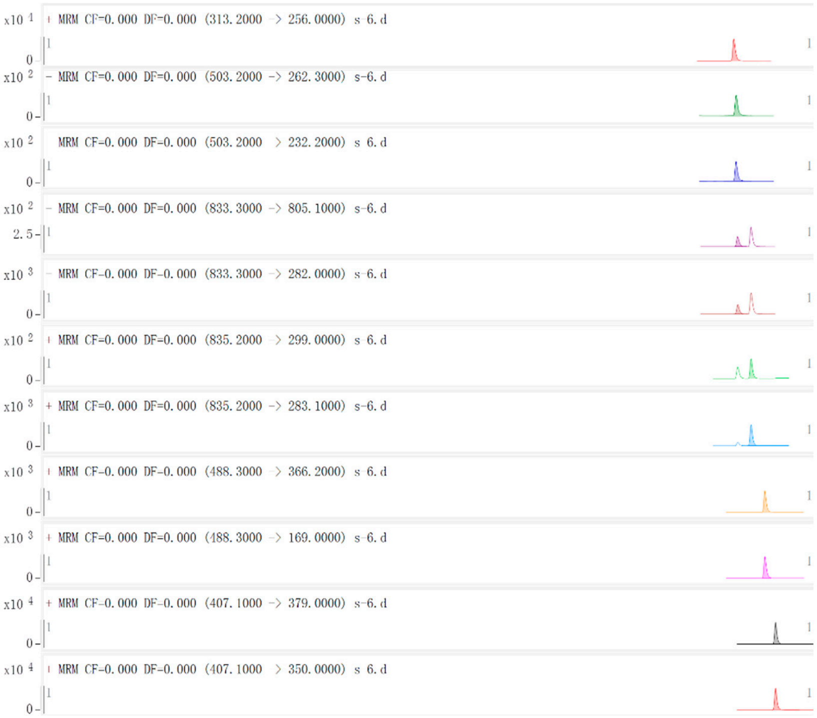

Figure S3-continued-17. The extract ion chromatograms (EICs) of transitions of all the PDE-5is.

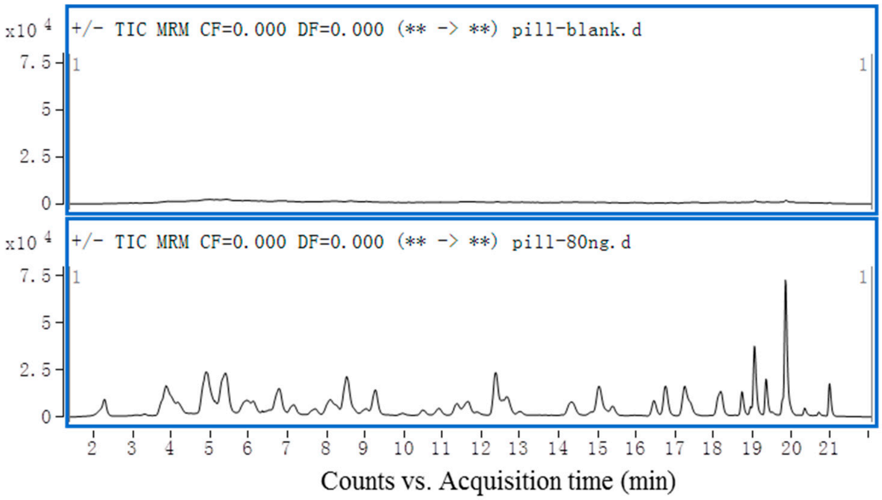

Figure S4. The total ion chromatograms (TIC) of blank pill matrix sample (upper) and spiked-in sample in pill matrix at concentration of 80ng/mL (lower).

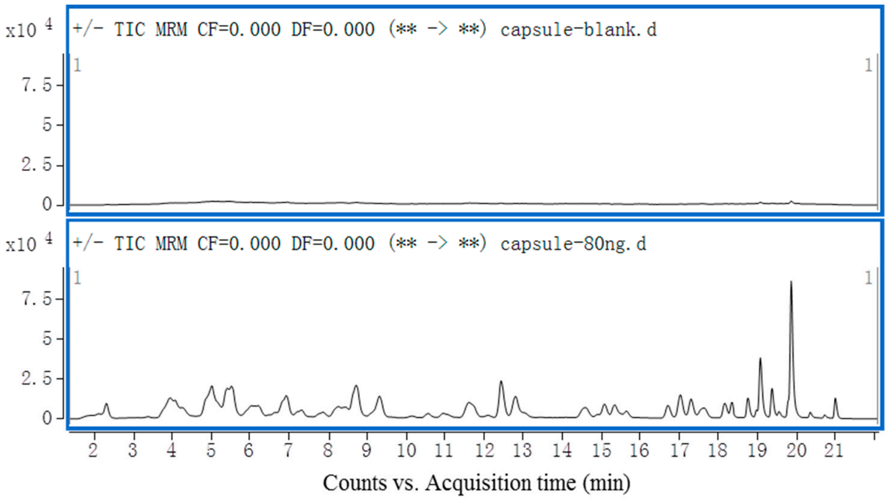

Figure S4-continued-1. The total ion chromatograms (TIC) of blank capsule matrix sample (upper) and spiked-in sample in capsule matrix at concentration of 80ng/mL (lower).

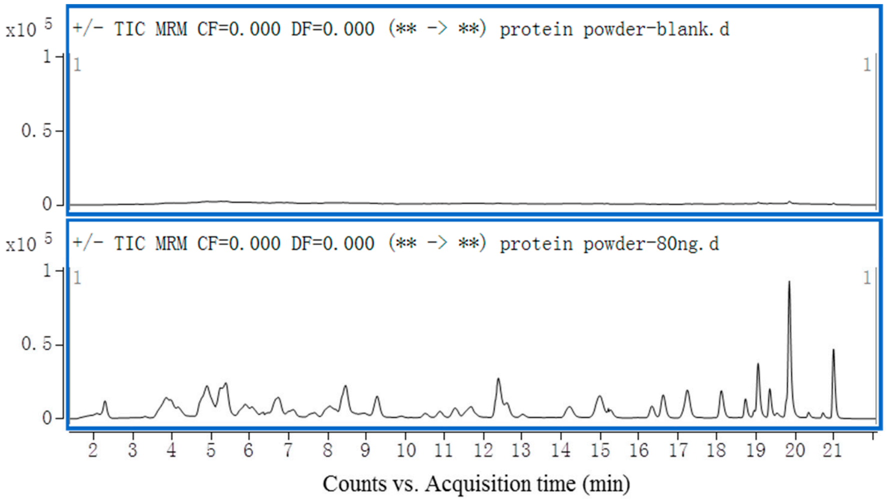

Figure S4-continued-2. The total ion chromatograms (TIC) of blank protein powder matrix sample (upper) and spiked-in sample in protein powder matrix at concentration of 80ng/mL (lower).

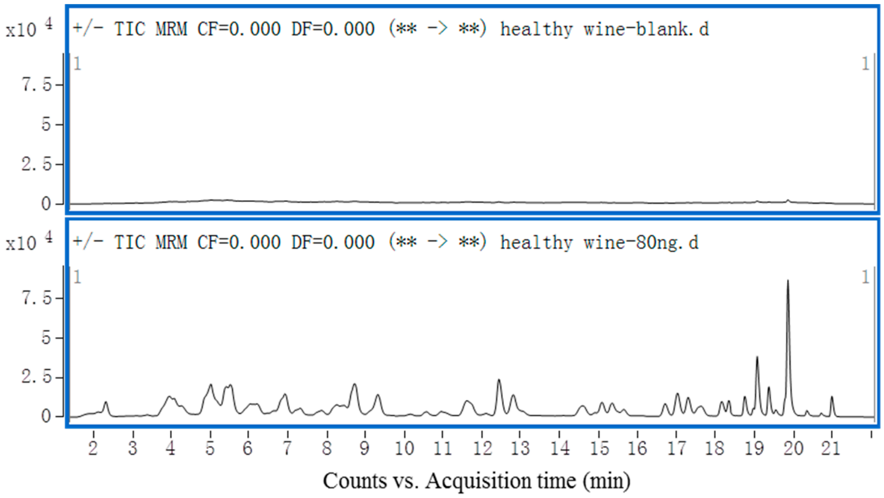

Figure S4-continued-3. The total ion chromatograms (TIC) of blank healthy wine matrix sample (upper) and spiked-in sample in healthy wine matrix at concentration of 80ng/mL (lower).

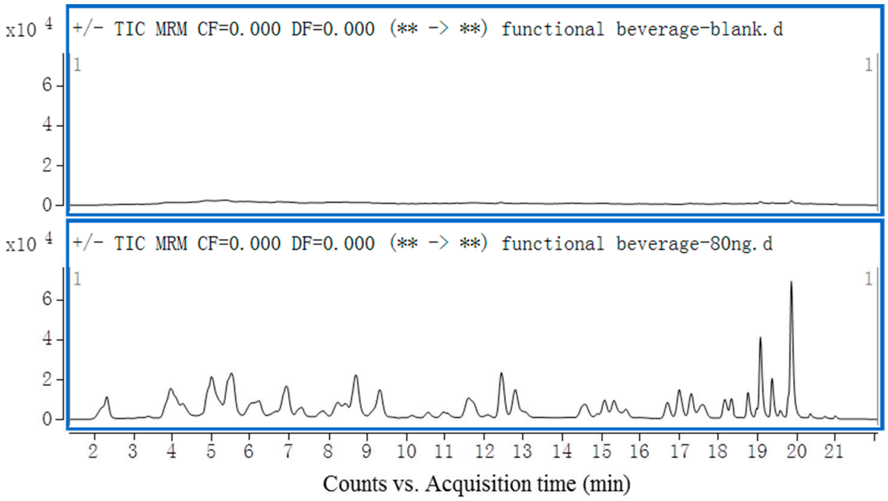

Figure S4-continued-4. The total ion chromatograms (TIC) of blank beverage matrix sample (upper) and spiked-in sample in healthy wine matrix at concentration of 80ng/mL (lower).
