# Supplementary material for: UPLC-MS/MS-Based Target Screening of 90 Phosphodiesterase Type 5 Inhibitors in 5 Dietary Supplements
Source: Molecules. 2024 Jul 30;29(15):3601. doi: 10.3390/molecules29153601 (PMC11313696; doi:10.3390/molecules29153601)
Supplement: Supplementary file 1 [file molecules-29-03601-s001.zip › Supplementary tables mentioned in the manuscript.pdf]

UPLC-MS/MS Based Target Screening of 90 Phosphodiesterase Type 5 Inhibitors in 5 Species Dietary Supplements

Shaoming Jin [1,†], Yaonan Wang [2,†], Xiao Ning [1], Tongtong Liu [1], Ruiqiang Liang [1] , Xinrong Pei [1,*] and Jin Cao 1,*

[1] National Institute for Food and Drug Control, Beijing 100050, China; myjackyming@126.com (S.J.); 15905171365@163.com (T.L.); nx200730079@163.com (X.N.); liangruiqiang1990@126.com (R.L.)

[2] School of Pharmaceutical Sciences, Capital Medical University, Beijing 100069, China; wangyaonan@ccmu.edu.cn

* Correspondence: peixinrong@nifdc.org.cn (X.P.), caojin@nifdc.org.cn (J.C.); Tel.: +86-010-6709-5264 (X.P.), +86-010-6709-5070 (J.C.)

† These authors contributed equally to this work.

Table S1. The Compound Name, Molecular Formula, CAS No, and Molecular Weight of 90 Phosphodiesterase type 5 inhibitors (PDE-5is)..1

Table S2. The detail parameters of established dMRM method..4

Table S1. The Compound Name, Molecular Formula, CAS No, and Molecular Weight of 90 Phosphodiesterase type 5 inhibitors (PDE-5is).

Compound Name	Molecular Formula	CAS No.	Molecular Weight
Sildenafil	C22H30N6O4S	139755-83-2	474.6
Tadalafil	C22H19N3O4	171596-29-5	389.4
Imidazosagatriazinone	C17H20N4O2	139756-21-1	312.4
Gendenafil	C19H22N4O3	147676-66-2	354.4
Acetil acid	C18H20N4O4	147676-78-6	356.4
Xanthoanthrafil	C19H23N3O6	1020251-53-9	389.4
Aminotadalafil	C21H18N4O4	385769-84-6	390.4
Chloropretadalafil	C22H19ClN2O5	171489-59-1	426.8
Piperiacetildenafil	C24H31N5O3	147676-50-4	437.5
Noracetildenafil	C24H32N6O3	949091-38-7	452.5
Carbodenafil	C24H32N6O3	944241-52-5	452.5
Pseudovardenafil	C22H29N5O4S	224788-34-5	459.6
Norneosildenafil	C22H29N5O4S	371959-09-0	459.6
N-Desethylvardenafil	C21H28N6O4S	448184-46-1	460.6
N-Desmethylsildenafil	C21H28N6O4S	139755-82-1	460.6
Acetildenafil	C25H34N6O3	831217-01-7	466.6
Hydroxyacetildenafil	C25H34N6O4	147676-56-0	482.6

Compound Name	Molecular Formula	CAS No.	Molecular Weight
Avanafil	$C_{23}H_{26}ClN_7O_3$	330784-47-9	484.0
Aildenafil	$C_{23}H_{32}N_6O_4S$	496835-35-9	488.6
Homosildenafil	$C_{23}H_{32}N_6O_4S$	642928-07-2	488.6
Vardenafil	$C_{23}H_{32}N_6O_4S$	224785-90-4	488.6
Thiosildenafil	$C_{22}H_{30}N_6O_3S_2$	479073-79-5	490.6
Thiohomosildenafil	$C_{23}H_{32}N_6O_3S_2$	479073-80-8	504.7
Hydroxyvardenafil	$C_{23}H_{32}N_6O_5S$	224785-98-2	504.6
Hydroxyhomosildenafil	$C_{23}H_{32}N_6O_5S$	139755-85-4	504.6
Udenafil	$C_{25}H_{36}N_6O_4S$	268203-93-6	516.7
Hydroxythiohomosildenafil	$C_{23}H_{32}N_6O_4S_2$	479073-82-0	520.7
Norneovardenafil	$C_{18}H_{20}N_4O_4$	358390-39-3	356.4
Nitrodenafil	$C_{17}H_{19}N_5O_4$	147676-99-1	357.4
Nortadalafil	$C_{21}H_{17}N_3O_4$	171596-36-4	375.4
Chlorodenafil	$C_{19}H_{21}ClN_4O_3$	1058653-74-9	388.8
Hydroxychlorodenafil	$C_{19}H_{23}ClN_4O_3$	1391054-00-4	390.9
N-Butyltadalafil	$C_{25}H_{25}N_3O_4$	171596-31-9	431.5
Desmethylcarbodenafil	$C_{23}H_{30}N_6O_3$	147676-79-7	438.5
Descarbonsildenafil	$C_{21}H_{30}N_6O_4S$	1393816-99-3	462.6
Dimethylacetildenafil	$C_{25}H_{34}N_6O_3$	1290041-88-1	466.6
Dithio-desmethylcarbodenafil	$C_{23}H_{30}N_6OS_2$	1333233-46-7	470.7
Oxohongdenafil	$C_{25}H_{32}N_6O_4$	1446144-70-2	480.6
N-Octylnortadalafil	$C_{29}H_{33}N_3O_4$	1173706-35-8	487.6
Dioxohongdenafil	$C_{25}H_{30}N_6O_5$	1609405-33-5	494.5
Hydroxythiovardenafil	$C_{23}H_{32}N_6O_4S_2$	912576-30-8	520.7
Cyclopentynafil	$C_{26}H_{36}N_6O_4S$	1173706-34-7	528.7
Propoxyphenyl thiohydroxyhomosildenafil	$C_{24}H_{34}N_6O_4S_2$	479073-90-0	534.7
Benzylsildenafil	$C_{28}H_{34}N_6O_4S$	1446089-82-2	550.7
Cinnamyldenafil	$C_{32}H_{38}N_6O_3$	1446089-83-3	554.7
Lodenafil carbonate	$C_{47}H_{62}N_{12}O_{11}S_2$	398507-55-6	1035.2
Propoxyphenylsildenafil	$C_{23}H_{32}N_6O_4S$	877777-10-1	488.6
Depiperazinothiosildenafil	$C_{17}H_{20}N_4O_4S_2$	1353018-10-6	408.5
Acetaminotadalafil	$C_{23}H_{20}N_4O_5$	1446144-71-3	432.4
2-Hydroxypropylnortadalafil	$C_{24}H_{23}N_3O_5$	1353020-85-5	433.5
Acetylvardenafil	$C_{25}H_{34}N_6O_3$	1261351-28-3	466.6
Propoxyphenyl hydroxyhomosildenafil	$C_{24}H_{34}N_6O_5S$	139755-87-6	518.6
Propoxyphenyl thioaildenafil	$C_{24}H_{34}N_6O_3S_2$	856190-49-3	518.7
Yohimbine	$C_{21}H_{26}N_2O_3$	146-48-5	354.4
Dapoxetine	$C_{21}H_{23}NO$	119356-77-3	305.4
N-Desethylacetildenafil	$C_{23}H_{30}N_6O_3$	147676-55-9	438.5
Desmethylthiosildenafil	$C_{21}H_{28}N_6O_3S_2$	479073-86-4	476.6

Compound Name	Molecular Formula	CAS No.	Molecular Weight
N-Boc-N-desethyl acetildenafil	C28H38N6O5	1246820-46-1	538.6
N-Ethyltadalafil	C23H21N3O4	1609405-34-6	403.4
O-Desethylsildenafil	C20H26N6O4S	139755-91-2	446.5
Pyrazole N-desmethylsildenafil	C21H28N6O4S	139755-95-6	460.6
Isobutylsildenafil	C23H32N6O4S	1391053-95-4	488.6
Sildenafil dimer impurity	C38H46N10O8S2	1346602-67-2	835.0
Vardenafil oxopiperazine	C21H26N6O5S	448184-58-5	474.5
Sildenafil N-oxide	C22H30N6O5S	1094598-75-0	490.6
Vardenafil N-oxide	C23H32N6O5S	448184-48-3	504.6
2-Hydroxyethylnortadalafil	C23H21N3O5	385769-94-8	419.4
Vardenafil acetyl analogue	C24H31N5O3	/	437.5
Vardenafil dimer	C38H46N10O8S2	1255919-03-9	835.0
Mirodenafil	C26H37N5O5S	862189-95-5	531.7
Mutaprodenafil	C27H35N9O5S2	1387577-30-1	629.8
Thioquinapiperfil	C24H28N6OS	220060-39-9	448.6
Aminosildenafil	C18H23N5O4S	319491-68-4	405.5
Desethylcarbodenafil	C22H28N6O3	1027192-92-2	424.5
Didescarbonsildenafil	C20H28N6O4S	466684-88-8	448.5
N-Phenylpropenyltadalafil	C30H24N4O4	2064212-00-4	504.5
N-Desethyl-N-methylvardenafil	C22H30N6O4S	224785-87-9	474.6
Thioaildenafil	C23H32N6O3S2	856190-47-1	504.7
Dichlorodenafil	C19H20Cl2N4O2	1446089-84-4	407.3
Piperazonifil	C25H34N6O4	1335201-04-1	482.6
Propoxyphenyl thiosildenafil	C23H32N6O3S2	479073-87-5	504.7
Propoxyphenyl thiohomosildenafil	C24H34N6O3S2	479073-88-6	518.7
Dithiodesethyl carbodenafil	C22H28N6OS2	1610830-81-3	456.6
Hydroxythioacetildenafil	C25H34N6O3S	1159977-47-5	498.7
Tadalafil dichloro impurity	C22H18Cl2N2O5	1598416-08-0	461.3
Sildenafil impurity 12	C25H34N6OS2	/	498.7
Demethylpiperaziny sildenafil sulfonic acid	C17H20N4O5S	1357931-55-5	392.1
Propoxyphenyl aildenafil	C24H34N6O4S	1391053-82-9	502.6
Sildenafil impurity 14	C24H32N6OS2	2146091-79-2	484.7
Propoxyphenylisobutyl aildenafil	C25H36N6O4S	1621873-33-3	516.7

Table S2. The detail parameters of established dMRM method.

Compound name	Precursor ion (m/z)	Product ion (m/z)	Fragmentary voltage (V)	Collision energy (V)	Polarity	Ret time (min)	Ret window
Sildenafil	475.2	283.1	170	46	+	6.4	2.3
	475.2	100.1*	170	30			
Tadalafil	390.2	268*	120	10	+	11.5	2.3
	390.2	135	120	26			
Imidazosagatriazinone	313.2	285. 1*	145	26	+	20.0	2.0
	313.2	256	145	34			
Gendenafil	355.2	327.1*	160	26	+	17.8	2.2
	355.2	285	160	34			
Acetil acid	357.2	329.1*	170	26	+	15.4	2.4
	357.2	285.1	170	34			
Xanthoanthrafil	390.2	151*	105	10	+	9.4	2.3
	390.2	107	105	74			
Aminotadalafil	391.1	269*	130	10	+	9.1	2.3
	391.1	204	130	66			
Chloropretadalafil	427.1	274	145	34	+	15.6	2.3
	427.1	135*	145	18			
Piperiacetildenafil	438.2	297.1	165	42	+	6.0	3.3
	438.2	98.1*	165	34			
Noracetildenafil	453.3	166	210	58	+	5.8	2.4
	453.3	97.1*	210	34			
Carbodenafil	453.3	339.1*	160	26	+	4.8	3.1
	453.3	311.1	160	38			
Pseudovardenafil	460.2	312.1	215	42	+	19.1	1.9
	460.2	151*	215	50			
Norneosildenafil	460.2	299.1	210	42	+	19.8	2.0
	460.2	282.9*	210	42			
N-Desethylvardenafil	461.2	312.1	195	38	+	5.9	1.9
	461.2	151*	195	58			
N-Desmethylsildenafil	461.2	311.1	180	34	+	6.5	2.6
	461.2	283*	180	42			
Acetildenafil	467.3	127.1	190	34	+	5.8	2.5
	467.3	111.1*	190	34			
Hydroxyacetildenafil	483.3	143.1	205	38	+	5.6	1.5
	483.3	127.1*	205	34			
Avanafil	484.2	375.1*	175	30	+	5.7	2.0
	484.2	155	175	46			
Aildenafil	489.2	113.1*	190	34	+	7.1	4.7
	489.2	99.1	190	38			
Homosildenafil	489.2	113.1*	180	30	+	6.5	4.7
	489.2	72.2	180	50			
Vardenafil	489.2	312.1	200	46	+	6.2	1.1
	489.2	151*	200	54			
Thiosildenafil	491.2	299	165	42	+	14.3	1.9
	491.2	100.1*	165	30			
Thiohomosildenafil	505.2	299	170	46	+	14.3	2.2
	505.2	113.1*	170	34			
Hydroxyvardenafil	505.2	312.1	190	46	+	6.5	1.8

Compound name	Precursor ion (m/z)	Product ion (m/z)	Fragmentary voltage (V)	Collision energy (V)	Polarity	Ret time (min)	Ret window
Hydroxyhomosildenafil	505.2	151*	190	54	+	6.9	2.7
	505.2	129.1	190	42			
	505.2	99.1*	190	42			
Udenafil	517.3	283*	200	50	+	7.9	3.1
	517.3	112.1	200	38			
Hydroxythiohomosildenafil	521.2	129.1	190	34	+	14.1	2.3
	521.2	99.1*	190	38			
Norneovardenafil	357.2	329.1	175	26	+	12.7	2.1
	357.2	151*	175	34			
Nitrodenafil	358.2	330.1*	160	26	+	19.5	1.9
	358.2	284.1	160	34			
Nortadalafil	376.1	254	105	10	+	9.6	2.5
	376.1	204*	105	70			
Chlorodenafil	389.1	361.1*	170	30	+	18.9	1.9
	389.1	285	170	38			
Hydroxychlorodenafil	391.2	313.1*	180	38	+	18.4	1.8
	391.2	285.1	180	34			
N-Butyltadalafil	432.2	310.1*	130	14	+	19.4	1.9
	432.2	135	130	30			
Desmethylcarbodenafil	439.2	339.1*	165	22	+	4.7	3.0
	439.2	311.1	165	34			
Descarbonsildenafil	463.2	311.1	165	34	+	5.7	2.9
	463.2	283.1*	165	42			
Dimethylacetildenafil	467.3	166	195	58	+	6.9	2.5
	467.3	127.1*	195	34			
Dithio-desmethylcarbodenafil	471.2	371.1*	165	22	+	15.6	2.4
	471.2	343.1	165	38			
Oxohongdenafil	481.3	297.1	165	50	+	12.1	2.8
	481.3	410.2*	165	34			
N-Octylnortadalafil	488.3	169	135	50	+	20.7	2.1
	488.3	366.2*	135	14			
Dioxohongdenafil	495.2	311.1	190	46	+	11.0	2.4
	495.2	127.1*	190	42			
Hydroxythiovardenafil	521.2	167*	215	70	+	11.3	2.3
	521.2	99.1	215	38			
Cyclopentynafil	529.3	461.2*	175	30	+	7.4	2.6
	529.3	98.1	175	42			
Propoxyphenyl thiohydroxyhomosildenafil	535.2	299.1	185	42	+	17.0	2.2
	535.2	99.1*	185	42			
Benzylsildenafil	551.2	377.1*	185	30	+	11.3	2.3
	551.2	134.1	185	42			
Cinnamyldenafil	555.3	117*	175	30	+	12.4	2.4
	555.3	115.1	175	80			
Lodenafil carbonate	518.3	230.2*	151	38	+	19.7	2.0
	518.3	112.2	151	38			
Propoxyphenylsildenafil	489.2	283.1	170	42	+	8.2	2.5
	489.2	166.2*	170	30			
Depiperazinothio	407.1	378.1	155	22	-	9.2	3.1

Compound name	Precursor ion (m/z)	Product ion (m/z)	Fragmentary voltage (V)	Collision energy (V)	Polarity	Ret time (min)	Ret window
sildenafil	407.1	314*	155	34			
Acetaminotadalafil	433.2	135	135	22	+	9.2	2.6
	433.2	204*	135	74			
2-Hydroxypropyl nortadalafil	434.2	312.1*	150	14	+	11.1; 11.9	2.7
	434.2	135	150	26			
Acetylvardenafil	467.3	151	195	54	+	5.3	2.5
	467.3	111.1*	195	34			
Propoxyphenyl hydroxyhomosildenafil	519.2	283	175	46	+	8.2	1.8
	519.2	99.1*	175	42			
Propoxyphenyl thioaildenafil	519.2	299*	160	38	+	18.0	1.8
	519.2	113.1	160	34			
Yohimbine	355.2	212	155	26	+	3.2	2.8
	355.2	144*	155	34			
Dapoxetine	306.2	157*	105	26	+	9.2	0.7
	306.2	127	105	62			
N-Desethylacetildenafil	439.2	166	160	58	+	5.0	2.3
	439.2	99.1*	160	34			
Desmethylthiosildenafil	477.2	327.1	175	30	+	14.1	2.4
	477.2	299.1*	175	42			
N-Boc-N-desethyl acetildenafil	539.3	439.2*	125	18	+	12.9	2.7
	539.3	99.1	125	46			
N-Ethyltadalafil	404.2	282.1*	120	10	+	13.7	3.4
	404.2	135	120	26			
O-Desethylsildenafil	447.2	299.1	155	34	+	8.5	1.2
	447.2	283*	155	30			
Pyrazole N-desmethylsildenafil	461.2	269	165	42	+	4.2	2.5
	461.2	100.1*	165	30			
Isobutylsildenafil	489.2	297.1*	170	46	+	8.2	2.0
	489.2	100.1	170	34			
Sildenafil dimer impurity	835.2	283.1*	225	78	+	19.9	2.9
	835.2	299	225	66			
Vardenafil oxopiperazine	475.2	312.1	160	42	+	9.8	2.2
	475.2	151*	160	58			
Sildenafil N-oxide	491.2	404.1	185	30	+	7.0	2.7
	491.2	99.1*	185	38			
Vardenafil N-oxide	505.2	477.2	130	22	+	6.0	2.3
	505.2	151*	130	74			
2-Hydroxyethylnor tadalafil	420.2	298.1*	120	10	+	9.9	3.3
	420.2	169	120	46			
Vardenafil acetyl analogue	438.2	98.1*	190	34	+	5.4	2.0
	438.2	151	190	54			
Vardenafil dimer	833.3	805.1	165	42	-	20.0; 20.4	2.4
	833.3	282*	165	66			
Mirodenafil	532.3	296.1*	220	46	+	11.7	3.5
	532.3	99.1	220	46			
Mutaprodenafil	630.2	312.1	160	46	+	10.0	2.3
	630.2	141.9*	160	30			
Thioquinapiperfil	449.2	204*	145	22	+	7.3	1.5
	449.2	186	145	42			

Compound name	Precursor ion (m/z)	Product ion (m/z)	Fragmentary voltage (V)	Collision energy (V)	Polarity	Ret time (min)	Ret window
Aminosildenafil	406.2	364.1*	165	26	+	12.1	3.0
	406.2	299.1	165	38			
Desethylcarbodenafil	425.2	339.1*	135	22	+	4.6	2.0
	425.2	311.1	135	34			
Didescarbonsildenafil	449.2	311.1	170	30	+	5.5	2.9
	449.2	283.1*	170	42			
N-Phenylpropenyl tadalafil	503.2	262.3*	150	20	-	20.9	2.2
	503.2	232.2	150	50			
N-Desethyl-N-methylva rdenafil	475.2	312.1	200	46	+	5.6	2.4
	475.2	151*	200	50			
Thioaildenafil	505.2	113.1*	170	34	+	14.5	2.2
	505.2	99.1	170	42			
Dichlorodenafil	407.1	379*	185	30	+	21.0	2.6
	407.1	350	185	34			
Piperazonifil	483.2	166	166	58	+	6.8	2.4
	483.2	436.2*	166	34			
Propoxyphenyl thiosildenafil	505.2	313.1	175	42	+	17.2	3.2
	505.2	299*	175	42			
Propoxyphenyl thiohomosildenafil	519.2	299*	175	42	+	17.2	1.8
	519.2	113.1	175	34			
Dithiodesethyl carbodenafil	457.2	371.1*	165	26	+	14.9	2.3
	457.2	343	165	38			
Hydroxythio acetildenafil	499.2	127.1	189	34	+	12.7	3.6
	499.2	143.1*	189	34			
Tadalafil dichloro impurity	461.1	204*	150	78	+	18.5	2.6
	461.1	135	150	22			
Sildenafil impurity 12	499.2	371.1	160	26	+	16.4	2.3
	499.2	343*	160	42			
Demethylpiperaziny sildenafil sulfonic acid	393.1	365.1*	175	30	+	5.5	2.3
	393.1	256	175	42			
Propoxyphenyl aildenafil	503.2	283*	185	38	+	9.0	1.8
	503.2	113.2	185	34			
Sildenafil impurity 14	485.2	371.1*	165	26	+	16.5	1.3
	485.2	343	165	38			
Propoxyphenylisobutyl aildenafil	517.3	297.3*	200	42	+	11.2	2.1
	517.3	113.1	200	34			

* : indicated this transition was used for quantification.
